# Strategies for effective high pressure germination or inactivation of *Bacillus* spores involving nisin

**DOI:** 10.1128/aem.02299-23

**Published:** 2024-09-23

**Authors:** Rosa Heydenreich, Alessia I. Delbrück, Clément Trunet, Alexander Mathys

**Affiliations:** 1 Sustainable Food Processing Laboratory, Institute of Food, Nutrition, and Health, Department of Health Science and Technology, ETH Zurich, Zurich, Switzerland; 2 Univ Brest, INRAE, Laboratoire Universitaire de Biodiversité et Écologie Microbienne, UMT ACTIA 19.03 ALTER’iX, Quimper, France; Anses, Maisons-Alfort Laboratory for Food Safety, Maisons-Alfort, France

**Keywords:** *Bacillus subtilis*, *Bacillus amyloliquefaciens*, high isostatic pressure, endospore, superdormant, inactivation, germination, nisin

## Abstract

The major challenge in employing high pressure (HP) at moderate temperature for sterilization is the remarkable resistance of bacterial spores. High isostatic pressure can initiate spore germination, enabling subsequent inactivation under mild conditions. However, not all spores could be triggered to germinate under pressure at temperatures ≤80°C so far. In this study, germination treatment combinations were evaluated for *Bacillus* spores involving moderate HP (150 MPa, 37°C, 5 min), very HP (vHP, 550 MPa, 60°C, 2.5 or 9 min), simple and complex nutrient germinants [L-valine, L-alanine, and tryptic soy broth (TSB)], nisin, and incubation at atmospheric pressure (37°C). The most effective combinations for *Bacillus subtilis* resulted in a reduction of culturable dormant spores by 8 log_10_ units. The combinations involved nisin, a nutrient germinant (L-valine or TSB), a first vHP treatment (550 MPa, 60°C, 2.5 min), incubation at atmospheric pressure (37°C, 6 h), and a second vHP treatment (550 MPa, 60°C, 2.5 min). Such treatment combination with L-valine reduced *Bacillus amyloliquefaciens* spores by only 2 log_10_ units. *B. amyloliquefaciens,* thus, proved to be substantially more HP-resistant compared to *B. subtilis*, validating previous studies. Despite combining different germination mechanisms, complete germination could not be achieved for either species. The natural bacteriocin nisin did seemingly not promote HP germination initiation under chosen HP conditions, contrary to previous literature. Nevertheless, nisin might be beneficial to inhibit the growth of HP-germinated or remaining ungerminated spores. Future germination experiments might consider that nisin could not be completely removed from spores by washing, thereby affecting plate count enumeration.

**IMPORTANCE:**

Extremely resistant spore-forming bacteria are widely distributed in nature. They infiltrate the food chain and processing environments, posing risks of spoilage and food safety. Traditional heat-intensive inactivation methods often negatively affect the product quality. HP germination–inactivation offers a potential solution for better preserving sensitive ingredients while inactivating spores. However, the presence of ungerminated (superdormant) spores hampers the strategy’s success and safety. Knowledge of strategies to overcome resistance to HP germination is vital to progress mild spore control technologies. Our study contributes to the evaluation and development of mild preservation processes by evaluating strategies to enhance the HP germination–inactivation efficacy. Mild preservation processes can fulfill the consumers’ demand for safe and minimally processed food.

## INTRODUCTION

So far, no high pressure (HP) process has achieved food sterilization at moderate temperatures (≤80°C). To implement such a gentle process, the resistance of bacterial spores needs to be overcome. Dormant spores are metabolically inactive and can withstand extreme conditions such as heat, dehydration, and chemical or other physical treatments. Spore-forming bacteria belong mainly to the classes *Bacilli* and *Clostridia* (1–3). Due to the spore’s resistance, many spores can survive current industrial non-thermal HP processing at 400–600 MPa and temperatures commonly ≤25°C (4). If the growth of surviving spores is not controlled by the food formulation or storage conditions, like energy-intensive refrigeration, spores may cause food spoilage or jeopardize food safety (5). Traditional heat sterilization (e.g., 121°C for certain minutes) for spore inactivation can deteriorate the food quality regarding its color, texture, or nutritional value (6–8). Less heat-intensive approaches are demanded to effectively inactivate spores to less compromise the food quality (6, 9, 10). A recently commercialized approach for spore inactivation, i.e., for sterilization, is so-called high pressure thermal processing at pressures of ≥600 MPa and temperatures of 90°C–121°C (6, 11, 12). This pressure–temperature combination enables more homogenous heating and shorter treatment times, thereby reducing the thermal load. To further reduce the thermal load and even better preserve food quality (13), spore inactivation processes at moderate temperatures (≤80°C) are of great interest.

A promising approach at moderate temperatures (≤80°C) would be an HP germination–inactivation process (10). The principle of this strategy is that HP can trigger germination, which transforms inactive dormant spores into physiologically active germinated spores. Germinated spores have lost their extreme resistance and are, thus, sensitive to a mild heat or HP inactivation treatment (14–16). Manifold HP treatment parameters have been tested over the last century in the range of 0.1–2,000 MPa and ≤80°C with treatment times from seconds to hours, various treatment repetitions, various buffers or food matrices, and various spore-forming species (17–22). The predominant HP germination mechanism depends on the HP level, temperature, and time. Shown for mostly *Bacillus*, moderate high pressure (mHP, 50–300 MPa, 30°C–50°C) triggers germination predominantly via activation of nutrient germinant receptors in the inner membrane. This leads to dipicolinic acid (DPA) release from the spore core. These two steps are similar to nutrient-stimulated germination as found in nature at atmospheric pressure. In the nutrient-stimulated pathway, small molecules such as specific amino acids, sugars, or purine nucleosides act as nutrient germinants, activating the germinant receptors (23). Very high pressure (vHP, 400–600 MPa) initiates germination predominantly via the release of DPA independently of germinant receptors (18, 24). The detailed nutrient or HP germination mechanisms are described elsewhere (25–27). With increasing vHP treatment temperature and time, completion of germination and outgrowth after the HP treatment become less likely. This means spores are increasingly inactivated under HP after their DPA release due to enzyme inactivation or membrane damage. Therefore, vHP at ≥600 MPa and high temperatures of >60°C can trigger unphysiological DPA release followed by rapid inactivation without requiring physiological germination steps. In comparison to vHP, mHP (50–300 MPa, 30–50°C) inactivates spores generally less efficiently once spores have released DPA under pressure (18, 19, 27, 28).

It should be noted that one pressure–temperature–time combination might predominantly cause germination or inactivation within a spore population and might be, therefore, theoretically considered as germination or inactivation treatment. The vHP treatments at 550 MPa and 60°C are considered as germination treatments in this study. This should highlight that completion of germination after the vHP treatment is possible for a fraction of spores.

For a successful germination–inactivation strategy, all spores must initiate germination in terms of DPA release. The DPA release is the critical step for the loss of heat and pressure resistance (28). Hence, vHP germination treatments aim primarily at initiating DPA release regardless of further physiological germination steps or direct inactivation. Complete germination initiation has not been achieved for all spore-formers yet, as a fraction of ungerminated spores remained, so-called superdormant spores (29). Superdormancy describes a decreased germination capacity of spores relative to the other spores within an isogenic spore population (29). Superdormancy has been observed for various germination stimuli, such as nutrients, mHP, or vHP, for example (26, 29). The properties of superdormant spores depend on the type and intensity of the triggered germination mechanism (15). Superdormancy seems to be caused by reversible and irreversible factors. Spores that were superdormant to one germination stimulus type, like a specific amino acid, were still susceptible to other types of germination stimuli (15, 30–33). Therefore, combining different germination stimuli might overcome superdormancy and achieve germination of all spores.

Nisin is a regulatory-approved natural antimicrobial preservative used in foods (34). It is a polypeptide of 34 amino acids produced by *Lactococcus lactis*. Its antimicrobial activity against Gram-positive bacteria relies on membrane pore formation (34, 35). As nisin and HP both target the inner spore membrane (36), a possible synergism in triggering germination or inactivation has been investigated. A positive effect of nisin on vHP germination (500 MPa, 40°C, 5 min) has been suggested in the presence of nutrients (37). From later studies at 500 MPa and 50°C, it remained open whether nisin promotes HP inactivation by facilitating DPA release or germination in the presence of nutrients (38, 39).

**Fig 1:**
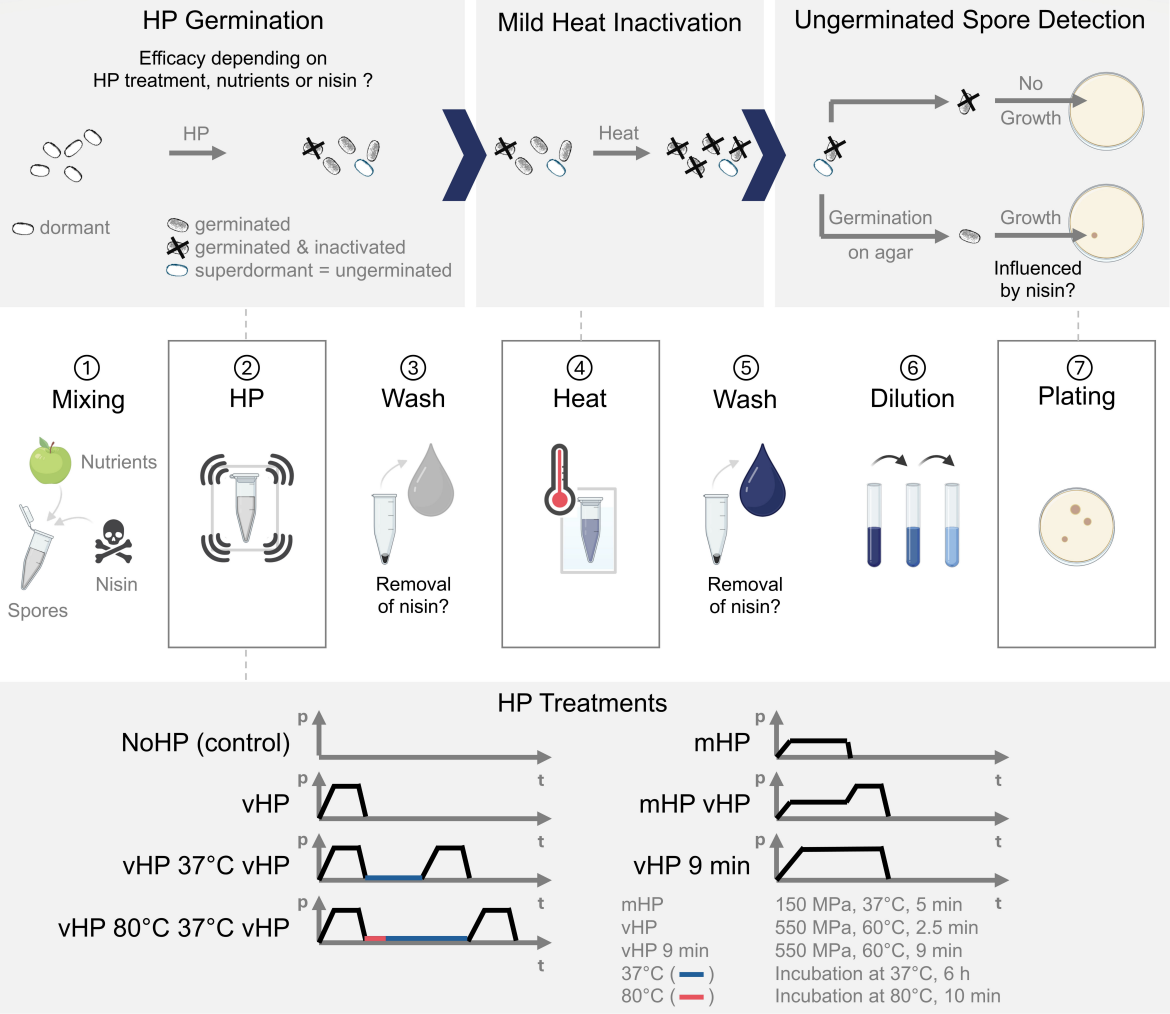
Graphical abstract of the experimental set-up of the HP spore germination–inactivation strategy and corresponding research questions. The experimental steps 2–5 were omitted or modified based on the research question. p: pressure; t: time.

This study evaluated the efficacy of germination trigger combinations for *Bacillus subtilis*, the model organism for germination (23), and for a particularly HP-resistant *Bacillus amyloliquefaciens* strain (40, 41). Tested parameters are visualized in Fig. 1. The parameters included nisin and nutrient germinants, such as L-valine, L-alanine, a mixture of L-asparagine, D-glucose, D-fructose, and potassium chloride (AGFK) (32), or tryptic soy broth (TSB). TSB contains various nutrient germinants (sugars and amino acids from digested proteins) (42). Spores were treated at mHP (150 MPa, 37°C, 5 min), vHP (550 MPa, 60°C, 2.5–9 min), or both mHP and vHP. Additional incubation at atmospheric pressure to trigger nutrient germination was tested too. The chosen pressures and temperatures are optimized conditions for mHP or vHP germination of *B. subtilis* (18, 26, 28, 43, 44). Despite previous studies on combining HP with other germination stimuli, the prediction of results at the chosen HP conditions was impossible. The reason is that HP–germination–stimuli combinations have led to contradictory results likely due to the complex interplay of influencing parameters (pressure, temperature, time, pH, suspending medium, nutrient germinant type and concentration, and bacterial species) (17, 45–48). Further research was thus required. A particular aim of the study was to clarify whether nisin plays a role in the initiation of HP germination or only promotes HP inactivation.

## RESULTS

### Choice of the nisin concentration

To choose the nisin concentration for HP experiments, growth inhibition was tested in a liquid medium at atmospheric pressure using different nisin concentrations (0.0025–2,500 IU/mL). Growth of *B. subtilis* was inhibited by concentrations of ≥250 IU/mL or 2,500 IU/mL as measured after 18 h or 3 days at 37°C, respectively. *B. amyloliquefaciens* was inhibited by 2,500 IU/mL nisin as measured after 18 h and 3 days at 37°C (File S1: Fig. S1).

**Fig 2:**
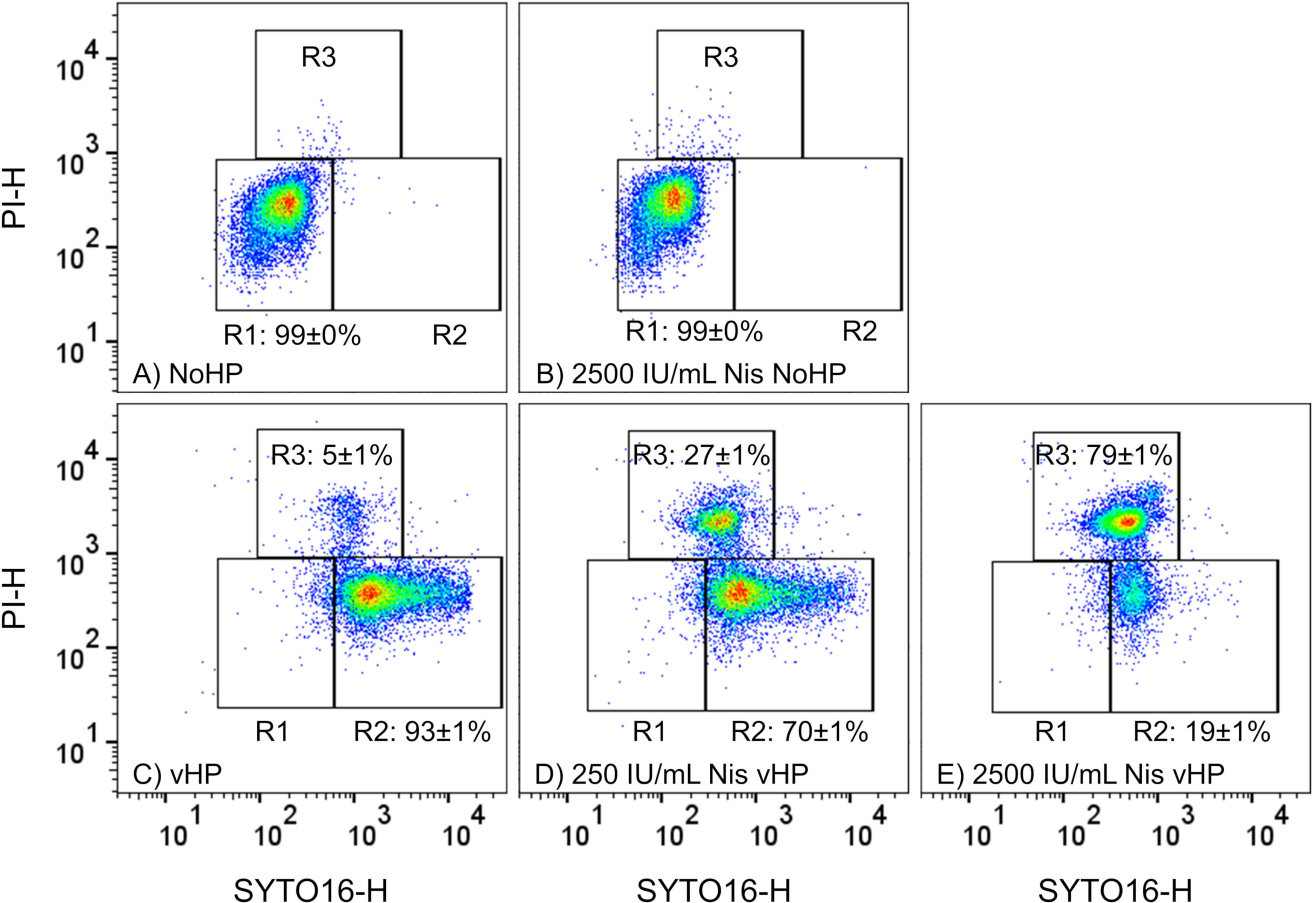
Effect of the nisin concentration on vHP-treated or dormant (NoHP) *B. subtilis* spores, analyzed by flow cytometry. Spores were non-HP-treated or vHP-treated in buffer (pH 7.0) at 550 MPa and 60°C for 2.5 min, washed, and kept on ice until analysis. In regions of certain fluorescence signal heights (-H), the following spore subpopulations appear: PI- and SYTO16-negative ungerminated spores in region “R1”; SYTO16-positive germinated spores with an intact inner membrane in region “R2”; and PI-positive germinated and presumably inactivated spores with membrane damage in region “R3.” Note that events in R1 of vHP-treated samples are not ungerminated spores as their number is below the detection limit after this treatment time (27). Percentages of events per region are shown as the mean ± standard deviation of three experimental replicates. Nis: nisin.

The effect of 250 and 2,500 IU/mL nisin on vHP treatments was further tested for *B. subtilis* spores using flow cytometry (FCM) and plate count (Table 1; Fig. 2). Before heat treatment (80°C, 20 min), the log_10_ reduction reflects the reduction of culturable spores. The remaining culturable spores can be germinated or ungerminated spores. The culturable spores were reduced due to inactivation of some spores by HP or nisin. After heat treatment, all germinated spores are inactivated and only ungerminated spores remain (Fig. 1). Therefore, the log_10_ reduction after heat treatment should reflect the reduction of culturable dormant spores, i.e., the extent of germination. Accordingly, without germination or inactivation treatment, no reduction of dormant spores should be observed. This was confirmed for the untreated control, as seen by a log_10_ reduction close to zero or which is within the range of a typical standard deviation for plate count (Table 1: NoHP). The log_10_ reduction close to zero additionally showed that the culturable dormant spore number was independent of a heat treatment at 80°C for the untreated dormant spore sample. A nisin concentration of 2,500 IU/mL did not induce germination (Fig. 2B) but inactivated dormant spores by 1 log_10_ unit (Table 1: "NoHP + 2,500 IU/mL Nis"). The inactivation by nisin was not visible in FCM plots (Fig. 2, see the similarity of plots A and B). Inactivation of dormant spores was observed even though the nisin-containing buffer had been removed by washing before plating. A vHP treatment alone of 2.5 min induced germination in a large fraction of spores seen by a reduction of >5 absolute log_10_ units after heat treatment (Table 1). Germinated spores were inactivated partially by vHP shown by 2.2 units log_10_ reduction before heat treatment (Table 1). This aligns with FCM plots, where the dormant spore population in “R1” disappeared due to germination; most spores were SYTO16-positive (“R2”), i.e. germinated; and a small propidium iodide (PI)-positive population (“R3”) was germinated and presumably inactivated (Fig. 2C). PI-positive spores were presumably inactivated, because the inner membrane permeabilization indicated by PI can be non-lethal depending on the treatment conditions (49). The higher the nisin concentration during the vHP treatments, the more spores were inactivated. This was suggested by a stronger log_10_ reduction of culturable spores before heat treatment with increasing nisin concentration (Table 1). At 2,500 IU/mL nisin, almost all HP-germinated spores seemed inactivated by nisin as indicated by a similar reduction before and after heat treatment (Table 1). Accordingly, the number of PI-positive spores (R3) increased with increasing nisin concentration while the number of HP-germinated spores with an intact membrane, indicated by SYTO16 fluorescence (R2), was reduced (Fig. 2D and E). The total number of HP-germinated spores (log_10_ reduction after heat treatment, Table 1) seemed not increased by the presence of nisin.

Lower nisin concentrations (e.g., 20 IU/mL) were previously found to enhance the effect of HP (inactivation) (38). However, a high nisin concentration of 2,500 IU/mL was chosen for further experiments to observe strong achievable effects in this mechanistic study. Hence, the high nisin concentration should lead to possibly clearer plate count results with or without nisin. Note that applied studies should consider the regulatory-approved nisin concentrations, which are significantly lower than 2,500 IU/mL (34, 38).

**TABLE 1:** Effect of the nisin (Nis) concentration on vHP-treated or dormant (NoHP) *B. subtilis* spores after washing, as analyzed by plate count

Treatment		log_10_ (*N/N*_0_)^*a*^ reduction of culturable spores; before heat treatment^*b*^ (mean ± SD, *n* = 3)	log_10_ (*N/N*_0_)^*a*^ reduction of culturable dormant spores; after heat treatment^*b*^ (mean ± SD, *n* = 3)
NoHP		+0.1 ± 0.0	0 (reference sample, *N* = *N*_*0*_)
NoHP	+2,500 IU/mL Nis	−0.9 ± 0.3	−0.9 ± 0.1
vHP^*c*^		−2.1 ± 0.3	−5.7 ± 0.2
vHP^*c*^	+250 IU/mL Nis	−3.6 ± 0.3	−5.8 ± 0.3
vHP^*c*^	+2,500 IU/mL Nis	−5.3 ± 0.2	−5.7 ± 0.1

^*a*^ Initial dormant spore concentration *N*_0_ after heat treatment: 10^9^ CFU/mL.

^*b*^ Heat treatment at 80°C for 20 min.

^*c*^ 550 MPa, 60°C, 2.5 min.

### Efficacy of combining different germination treatments of *B. subtilis*

**Fig 3:**
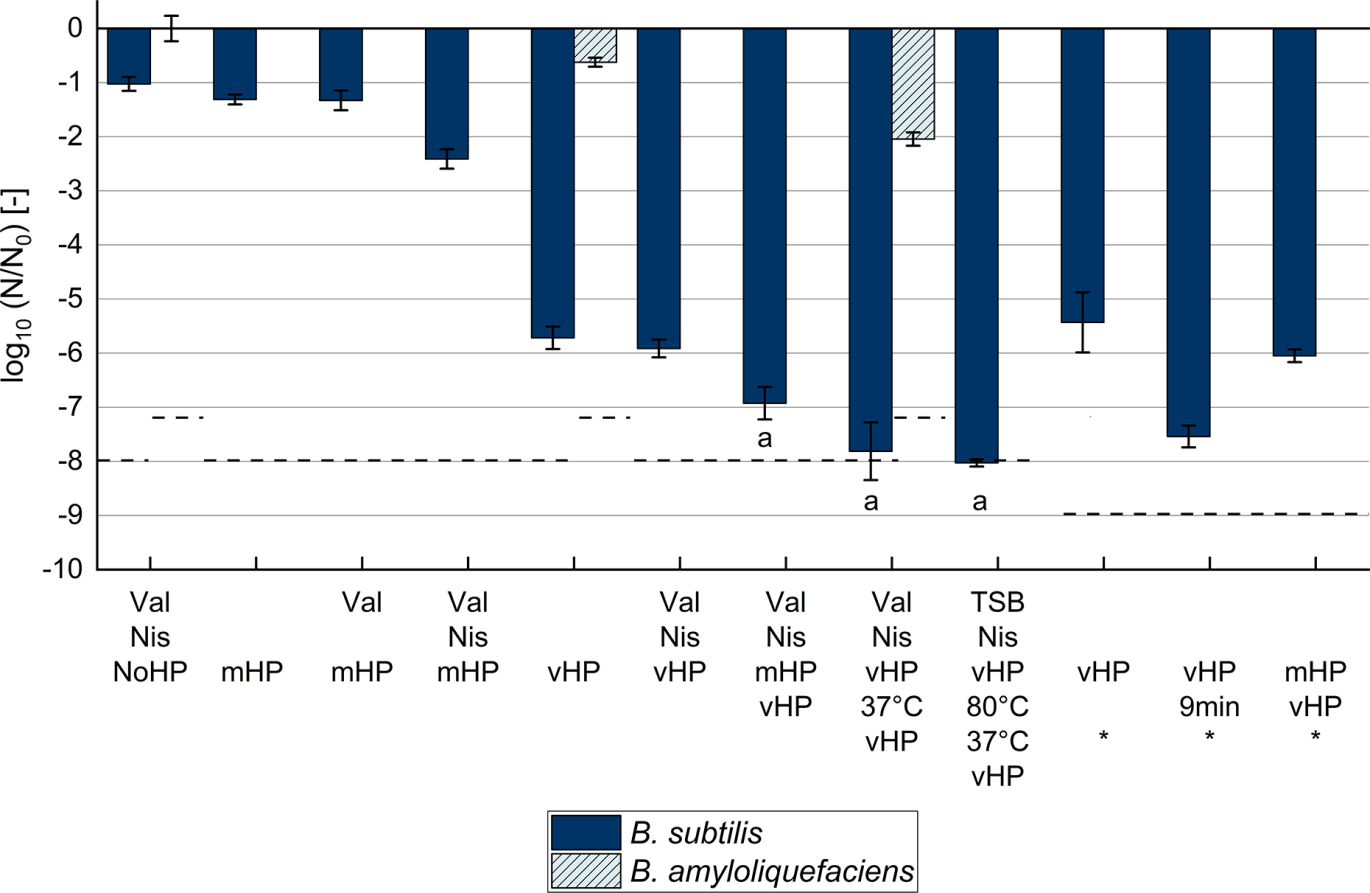
Reduction of culturable dormant spores (***N***) after different germination treatments, washing, and heat treatment (80°C, 20 min), as analyzed by plate count. Dormant spores were treated without pressure (NoHP: kept on ice), at mHP (150 MPa, 37°C, 5 min), or at vHP [550 MPa, 60°C, 2.5 min (or 9 min for “vHP 9 min*”)] with or without 100 mM L-valine, TSB, or 2,500 IU/mL nisin at initial dormant spore concentrations (***N*_0_**) of 10^9^ CFU/mL (*B. subtilis*) or 10^8^ CFU/mL (*B. amyloliquefaciens*). “mHP vHP” means an mHP treatment was immediately followed by a water batch switch of ~30 s for a subsequent vHP treatment. “37°C” means that samples were incubated at 37°C for 6 h for nutrient germination before a second vHP treatment. “80°C” means heat activation for improved nutrient germination at 80°C for 10 min. Error bars present standard deviations of independent experiments (*n* ≥ 3). ^*^ Data from spore batch 2 (*N*_0_ of 10^10^ CFU/mL, plated without washing after heat treatment). ^- - -^Detection limit. ^a^Estimated values (*N* < 100 CFU/mL). Treatments of *B. subtilis* at mHP or vHP with nisin and L-alanine instead of L-valine are not shown but were similar (File S1: Table S1). Nis: nisin; Val: L-valine

To evaluate HP germination strategies for the reduction of dormant *B. subtilis* spores, spores were subjected to different treatment combinations of HP with other potential germination promotors (Fig. 3). For the interpretation of the results below, it should be noted that the ungerminated spores of the untreated NoHP sample are termed dormant, as this sample represents the initial dormant population. This initial dormant population contains spores, that remain dormant or ungerminated after a (HP) germination treatment. These remaining ungerminated spores are termed superdormant to highlight that they resisted a germination treatment (Fig. 1).

Germination treatments at mHP (150 MPa, 37°C, 5 min) resulted in the least effective reduction of −1 log_10_ unit (Fig. 3). The combination of mHP and valine (or alanine) did not further reduce culturable dormant spores (Fig. 3). The addition of nisin to mHP treatments with valine promoted the reduction by 1 additional log_10_ unit, in contrast. More effective log_10_ reductions of −5 to −6 were measured for treatments at vHP (550 MPa, 60°C) for 2.5 min alone (Fig. 3), vHP combined with valine or the AGFK nutrient mix (File S1: Table S2), or vHP combined with valine and nisin (Fig. 3). Different spore batches seemed to behave similarly under vHP indicated by similar reductions under vHP alone for 2.5 min obtained for two spore batches, independent of the spore concentration (10^9^ or 10^10^ CFU/mL) (Fig. 3; File S1: Table S2). A clear benefit of combining mHP with vHP of 2.5 min could not be observed. However, with additional valine and nisin, a stronger reduction of −7 log_10_ units was measured for the “Val Nis mHP vHP” treatment (Fig. 3). The reduction was similar after vHP alone for a longer treatment time of 9 min, which corresponds to the same total treatment time as of the “Val Nis mHP vHP” treatment (Fig. 3). After only one HP and heat treatment, the remaining ungerminated spores were capable of nutrient germination indicated by growth on agar plates, i.e., log_10_ reductions above the detection limit (Fig. 3). Hence, samples with nutrients (valine or TSB) were incubated at 37°C for 6 h at atmospheric pressure after HP treatment to allow these remaining superdormant spores to undergo nutrient germination as on agar plates. A second vHP treatment followed to increase their germination likelihood. A heat activation (80°C, 10 min) before incubation at 37°C was tested too, as it can promote nutrient germination (50, 51). The treatments with two vHP treatments and incubation at 37°C were most effective in the reduction of culturable dormant spores. Reductions were close to the detection limit of −8 log_10_ units independent of heat activation or the nutrient germinant type (valine or TSB as complex medium with sugars and amino acids). Still, complete germination was not achieved. Estimated <40 out of 10^9^ spores/mL had not germinated during the germination treatments, therefore survived the heat treatment, and could germinate and grow on agar plates.

Growth was only observed after spore washing. Growth seemed not possible in unwashed, nisin-containing *B. subtilis* “TSB Nis vHP 80°C 37°C vHP” samples. This was indicated by phase dark (germinated) spores and a lack of vegetative cells under the microscope after incubation for 14 days at 37°C. The absence of growth was expected from growth inhibition for at least 3 days by 2,500 IU/mL nisin alone at atmospheric pressure (File S1: Fig. S1).

### Effect of nisin on HP-treated spores

Nisin could have influenced plate count results (Fig. 3) by promoting HP germination or by inhibiting growth on agar plates. To differentiate between these two effects, further plate count and FCM experiments were done.

**Fig 4:**
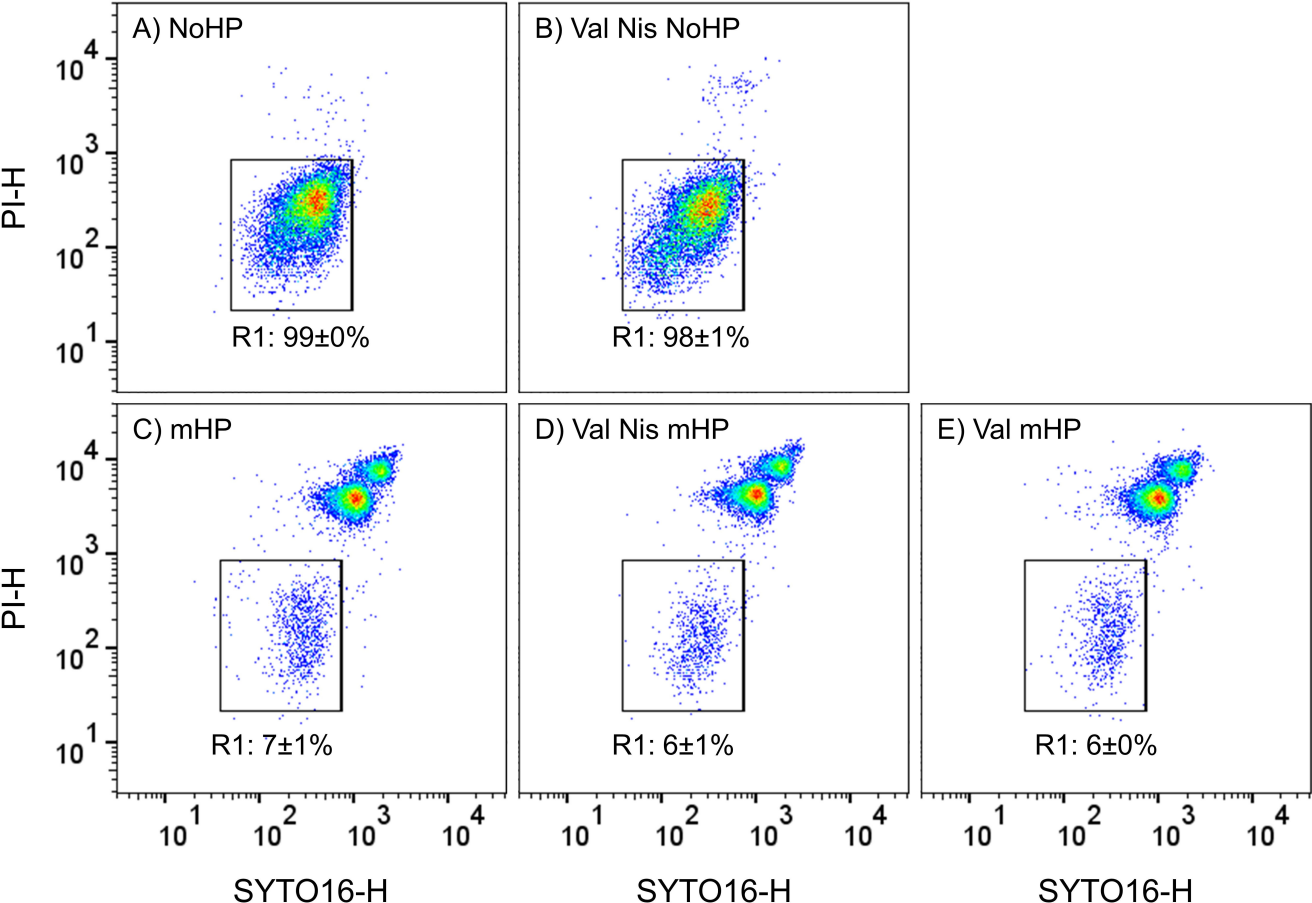
Flow cytometric quantification of ungerminated *B. subtilis* spores in mHP-treated or dormant spore (NoHP) samples after washing and heat treatment (80°C, 20 min). Spores were treated in buffer (pH 7.0) at 150 MPa and 37°C for 5 min (mHP) with or without 100 mM L-valine or 2,500 IU/mL nisin. In regions of certain fluorescence signal heights (-H), the following spore subpopulations appear: PI- and SYTO16-negative, ungerminated spores in region “R1”; SYTO16-positive, germinated spores with an intact inner membrane in region “R2”; and PI-positive, germinated, and presumably inactivated spores with membrane damage in region “R3.” The samples were exactly the same as those used for plate count analysis shown in Fig. 3. Percentages of events per region are shown as the mean ± standard deviation of three experimental replicates. Val: L-valine; Nis: nisin.

**Fig 5:**
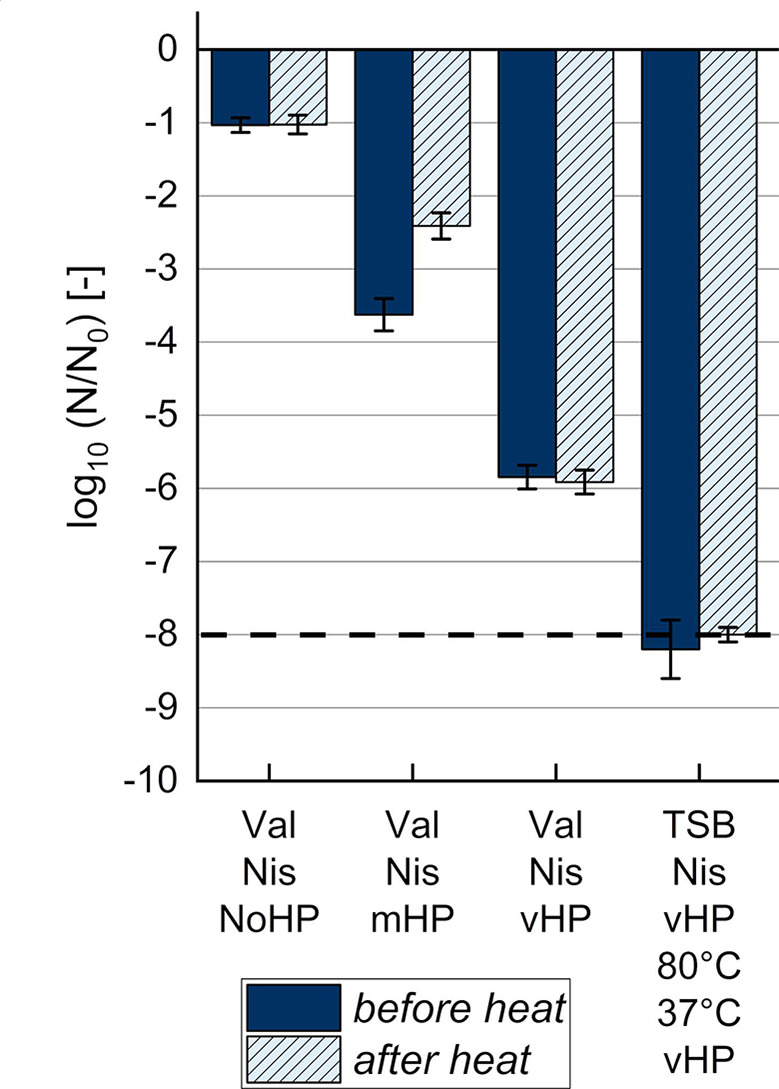
Influence of heat treatment (80°C, 20 min) on the reduction of culturable dormant *B. subtilis* spores (***N***) in nisin-containing samples, which were washed and plated before or after heat treatment (80°C, 20 min). Dormant spores were treated without pressure (NoHP: kept on ice), at mHP of 150 MPa and 37°C for 5 min, or at vHP of 550 MPa and 60°C for 2.5 min, with 100 mM L-Val in buffer (pH 7.0) or with TSB, and 2,500 IU/mL Nis at an initial dormant spore concentration (***N*_0_**) of 10^9^ CFU/mL. “80°C” and “37°C” mean that samples were heat-activated at 80°C for 10 min and incubated at 37°C for 6 h for nutrient germination before a second vHP treatment. Error bars present standard deviations of independent experiments (*n* ≥ 3). ^- - -^Detection limit. Val: L-valine; Nis: nisin.

Nisin inactivated dormant (NoHP) spores without inducing germination — independent of the presence of valine (Table 1; Fig. 3: “Val Nis NoHP”; Figure 4A and B). After an mHP or “Val mHP” treatment, the number of ungerminated spores was similar for FCM (6%–7%; Fig. 4) and plate count (~5% = −1.3 log_10_ units; Fig. 3). However, with nisin, ~0.4% ungerminated spores would have been expected from plate count (~0.4% = −2.4 log_10_ units, “Val Nis mHP”; Fig. 3). This conflicted with the percentage of ungerminated phase bright spores under the microscope (~1%–10%) or the 6% ungerminated spores measured by FCM (Fig. 4D). Consequently, nisin had seemingly not been completely removed by washing and promoted growth inhibition instead of mHP germination initiation, leading to the stronger log_10_ reduction. The growth inhibition of mHP-treated spores by nisin was influenced by the heat treatment before plate count (Fig. 5). More colonies, or a less negative log_10_ reduction, were detected after the heat treatment. This was unexpected as the heat treatment usually leads to fewer colonies, or a more negative log_10_ reduction, due to the inactivation of culturable HP-germinated spores [see Table 1: vHP, or references (14, 26, 49, 52)]. Interestingly, heat treatment did not affect the plate count of other samples with nisin, such as “Val Nis NoHP,” “Val Nis vHP,” or “TSB Nis vHP 80°C 37°C vHP” (Fig. 5). It suggests that, after these vHP treatments, all remaining viable germinated spores had been inactivated also without heat treatment due to inactivation by nisin.

**Fig 6:**
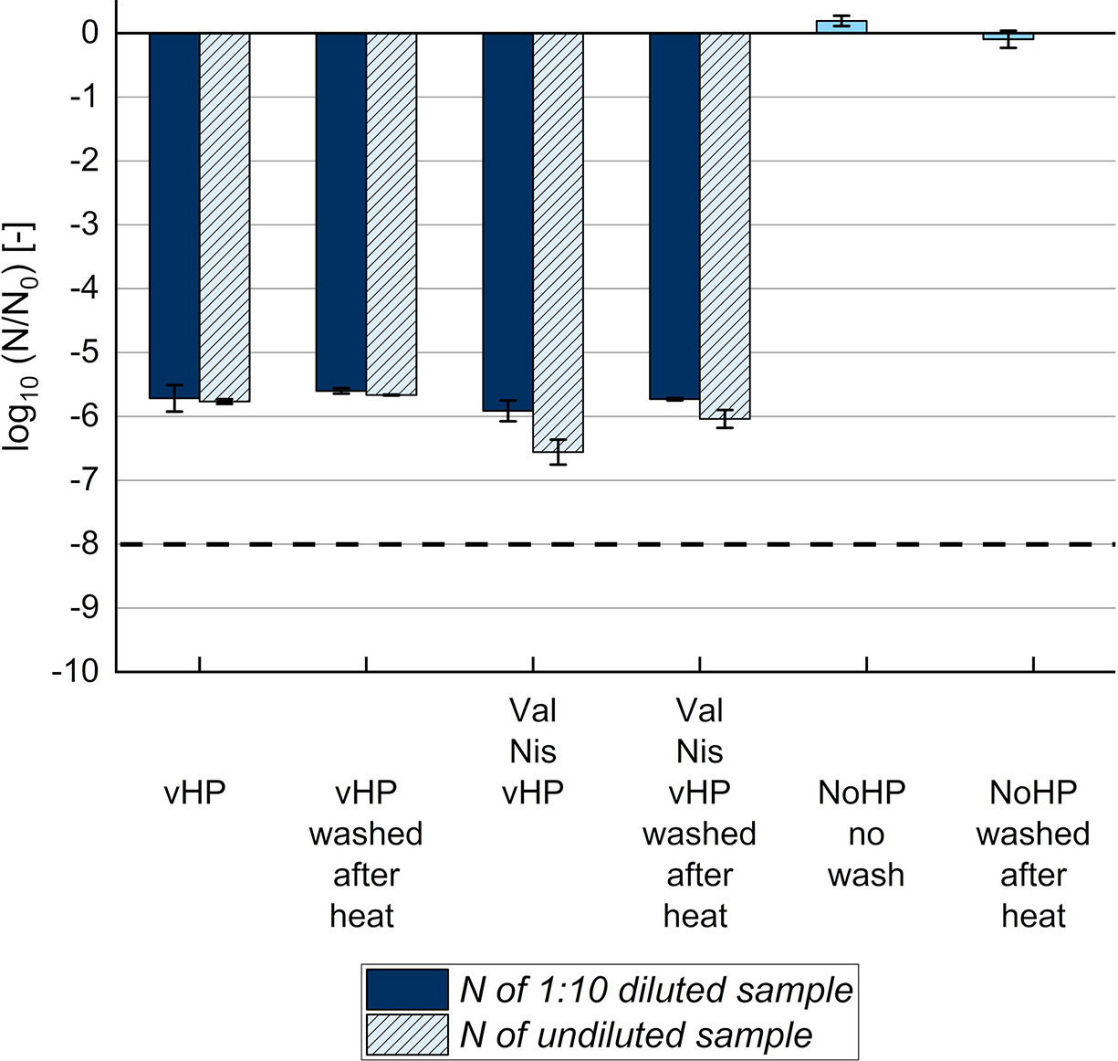
Influence of sample dilution or washing after heat treatment on the reduction of culturable dormant *B. subtilis* spores. Dormant spores were treated in buffer (pH 7.0) without pressure (NoHP: kept on ice) or at vHP of 550 MPa and 60°C for 2.5 min, with or without 100 mM L-valine and 2,500 IU/mL nisin at an initial dormant spore concentration (***N*_0_**) of 10^9^ CFU/mL. Samples were (i) washed 3× before the heat treatment (80°C, 20 min) by three cycles of centrifugation and supernatant exchange with ACES buffer at pH 7.0 (standard procedure), (ii) washed 3× before and 1× after heat treatment (“washed after heat”), or (iii) not washed at all (“no wash”). For the calculation of the culturable ungerminated spore concentration (***N***), the undiluted or 10× (1:10) diluted vHP samples were plated on agar. For NoHP samples with nisin, *N* was similar for the plated dilutions, resulting in similar log_10_ reductions (e.g., for NoHP with nisin and 100 mM L-alanine after heat treatment and standard washing procedure: 1:10^5^ dilution: −1.1 ± 0.1 log_10_ units; 1:10^6^ dilution: −1.1 ± 0.1 log_10_ units). Error bars present standard deviations of independent experiments (*n* ≥ 3). ^- - -^Detection limit. Val: L-valine; Nis: nisin.

For vHP samples, nisin seemed to have a dilution-dependent influence on growth, as the log_10_ reduction depended on the sample dilution before plate count (Fig. 6). A 1:10 sample dilution means that 10× less colonies should grow on agar plates compared to the undiluted sample. Log_10_ reductions should be independent of the dilution, as seen for vHP samples without nisin (Fig. 6), because the dilution factor is respected in the calculations [File S1: equations (1) and (2)]. However, only ~2× instead of 10× fewer colonies compared to the undiluted sample were counted after 1:10 dilution of “Val Nis vHP” samples with nisin. Therefore, a 0.7-unit stronger log_10_ reduction compared to the 1:10 diluted sample was obtained for undiluted “Val Nis vHP” samples (Fig. 6). The raw data set contains several more examples of the dilution-dependent CFUs for vHP treatments with nisin (e.g., “2,500 IU/mL Nis vHP—after heat treatment,” “2,500 IU/mL Nis vHP—before heat treatment,” and “Ala Nis vHP—after heat treatment”). To not overestimate log_10_ reductions of culturable dormant spores, results in Fig. 3 were consequently calculated based on the more diluted samples, if applicable. An influence of the dilutions was still observed after 1× additional washing after the heat treatment in vHP samples with nisin (Fig. 6). In contrast, results of vHP or NoHP samples without nisin were similar with or without additional washing after the heat treatment (Fig. 6). By washing after the heat treatment, it was tested if heat promotes the detachment of nisin from spores and thereby a transfer of nisin to the buffer, which can subsequently be removed by washing. Washing under acidic conditions (pH 2.5) before heat treatment might completely remove nisin due to an improved solubility of nisin at low pH (53). However, acidic washing of mHP or NoHP samples with nisin in our study seemed to be better but still did not completely remove nisin. Despite acidic washing, dormant (“Val Nis NoHP”) spores were still reduced by 0.8 ± 0.0 log_10_ units (File S1: Fig. S2).

### Comparison of germination experiments between *B. subtilis* and *B. amyloliquefaciens*

To assess species variability, *B. amyloliquefaciens* spores were HP-treated. While vHP (550 MPa, 60°C) alone of 2.5 min reduced dormant *B. subtilis* spores by >5 log_10_ units, *B. amyloliquefaciens* spores were only reduced by <1 log_10_ unit (Fig. 3). After a 20-min vHP treatment, *B. amyloliquefaciens* spores were only reduced by <2 log_10_ units (File S1: Fig. S3). FCM analysis resulted in ungerminated spores in the same order of magnitude as plate count, suggesting that these vHP treatments induced partial germination (File S1: Fig. S3 and S4). The treatment with two vHP treatments, nisin, valine, and incubation at 37°C was one of the most effective treatments for *B. subtilis* but led to a poor reduction of 2 log_10_ units for *B. amyloliquefaciens* (Fig. 3: “Val Nis vHP 37°C vHP”).

Dormant *B. amyloliquefaciens* spores were not clearly reduced by nisin and valine in the absence of HP, whereas *B. subtilis* spores were reduced by 1 log_10_ unit under the same conditions (Fig. 3: “Val Nis NoHP”). *B. amyloliquefaciens* seemed generally more resistant to nisin than *B. subtilis* indicated by the growth curves at lower nisin concentrations (File S1: Fig. S1: 25 or 250 IU/mL).

The washing procedure led to a slight spore loss for both *Bacillus* species seen by a slightly positive log_10_ reduction for the unwashed NoHP samples compared to the 3× washed NoHP sample after heat treatment (*B. subtilis*: see Fig. 6: “NoHP no wash”; *B. amyloliquefaciens*: 0.4 ± 0.3 log_10_ units). For *B. amyloliquefaciens*, more spores were lost on average because they pelleted less, as observed by the eye, leading to more spores in the discarded supernatant. *B. amyloliquefaciens* spores stuck better to the tube wall and formed a less compact spore pellet compared to *B. subtilis*, as observed before (50). Another difference between the two species was their sporulation conditions. The sporulation protocol of *B. subtilis* led to a poor dormant spore purity for *B. amyloliquefaciens* and biofilm formation, which prevented the harvest of single spores.

## DISCUSSION

### Influence of nisin on HP treatments

Nisin likely promoted inactivation instead of promoting HP germination at 150 MPa and 37°C (mHP) or 550 MPa and 60°C (vHP) in our study (Fig. 4 to 6). This finding contradicts the study of Black et al. (37) who observed a by 1–2 log_10_ units increased HP germination in the presence of nisin and nutrients using plate count. Black et al. treated *B. subtilis* spores at a comparable vHP level of 500 MPa at 40°C for 5 min with a similar nutrient concentration (100 mM L-alanine) but a lower nisin concentration (500 IU/mL nisin). An effect of nisin on growth inhibition rather than on germination in their study cannot be excluded, especially because the removal of the nisin-containing buffer before plate count was not reported. The different observations compared to our results might also been related to the different treatment temperatures. Without nutrients, vHP germination seemed unaffected by nisin in their study, similar to our results (Table 1).

Spore inactivation by nisin requires germination initiation (54). For nutrient germination of *Bacillus* spores, it was observed that (i) nisin did not influence the germination time (55) and (ii) nisin irreversibly inhibited the growth quickly after germination initiation (within <5 min) (35, 54). The bactericidal mode of action involved binding of nisin to lipid II in the inner membrane (35). Growth was inhibited by nisin in the germinating spores by preventing the establishment of oxidative metabolism and of a membrane potential due to pore formation in the inner membrane (54). Inner membrane damage to vHP-germinated spores by nisin can be confirmed in our study by PI staining (Fig. 2). These previous findings imply for the NoHP or HP-treated samples with nisin that the remaining ungerminated spores were likely inactivated by nisin only upon nutrient germination on agar (Table 1; Fig. 3 to 6).

**Fig 7:**
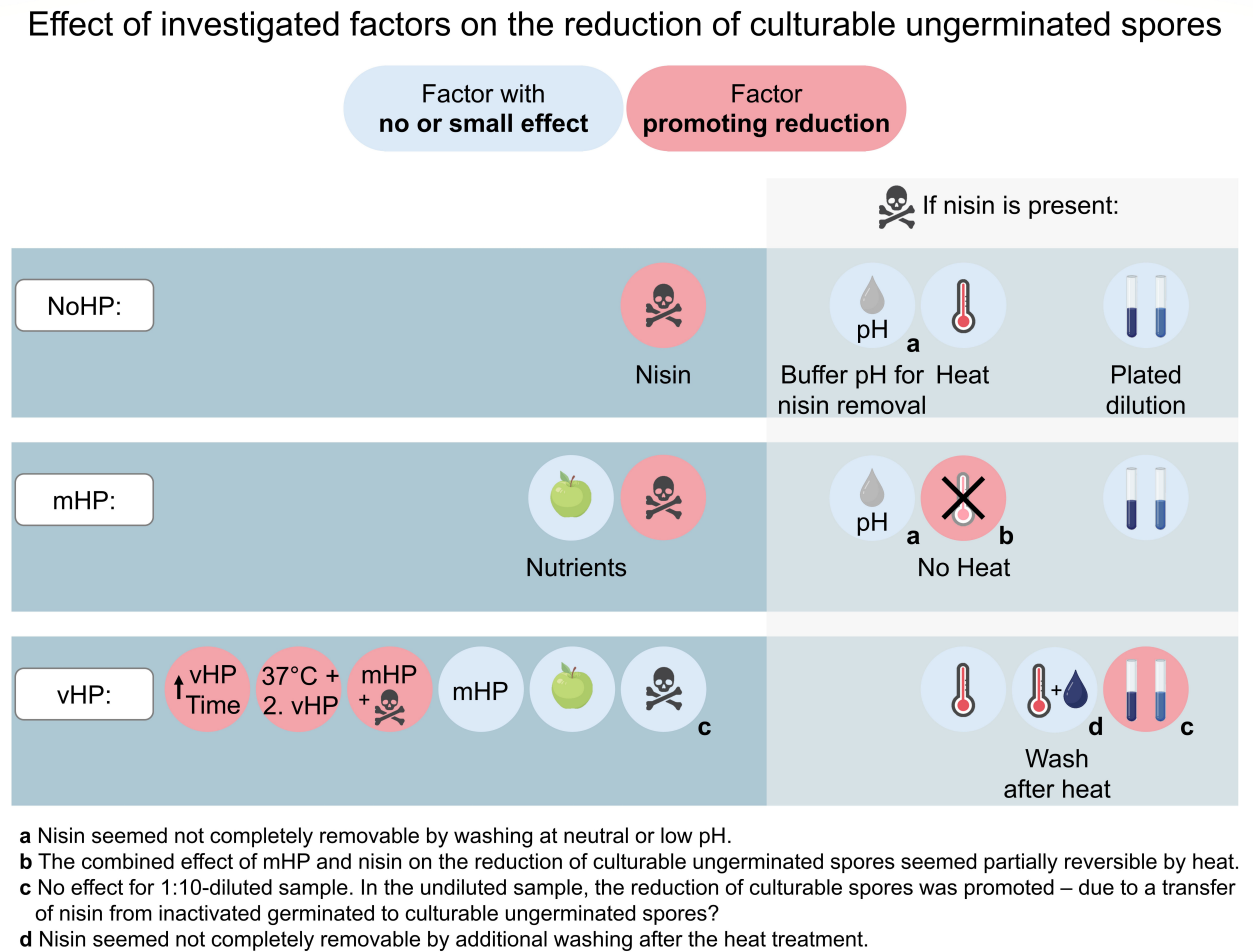
Summary of factors influencing the reduction of culturable ungerminated spores. The factors were combined with non-high-pressure-treated *B. subtilis* spores (NoHP) or with mHP (150 MPa, 37°C, 5 min) or vHP (550 MPa, 60°C, 2.5 min) treatments. The factor “37°C + 2. vHP” corresponds to a vHP treatment followed by incubation at 37°C and a second vHP treatment in the presence of nutrients and nisin. Refer to Fig. 1 for an overview of tested parameters. Refer to the Results and Discussion to read whether a factor promoting reduction reduced the number of culturable ungerminated spores by promoting HP germination or inhibiting their growth.

Spore inactivation by nisin on agar seemed possible as nisin was seemingly bound to the spores in a manner that nisin could not be removed by washing. Accordingly, neither washing before, nor additional washing after heat treatment, nor washing under acidic conditions could remove nisin completely (Fig. 6 and Fig. 7; File S1: Fig. S2). Washing of spores was done because others have washed *Paenibacillus* or *Terribacillus* spores once with phosphate-buffered saline (PBS) buffer for nisin removal after HP treatment with 50 IU/mL nisin (56). Yet, others washed “extensively” to lower the nisin concentration below the inhibitory concentration in a *Bacillus anthracis* spore suspension without stating the detailed washing protocol (54). In another study, however, nisin was not removed by washing with MilliQ water (55). These dormant spores were incubated with a lethal concentration of nisin and then washed to intentionally “pre-coat” spores with nisin before nutrient germination (55).

The dilution-dependent log_10_ reduction of vHP samples with nisin (Fig. 6 and 7) might have been an effect of nisin being carried over by germinated spores on agar plates. The spores on agar plates were mostly germinated non-culturable (~10^8^ spores) and few culturable (≤260) spores in the undiluted vHP or “Val Nis vHP” samples (Fig. 6). We hypothesize that the higher the sample dilution, the lower the total number of spores, the less carried-over nisin, the less the cultivability of ungerminated spores might have been affected on agar plates. This might explain why an influence of the dilution was not noticed for mHP or NoHP samples with nisin, as these samples were more diluted (by 1:≥10^3^) before plating.

Treatment at mHP (150 MPa, 37°C, 5 min) in our study might have caused modifications that promoted nisin binding. This might explain the stronger log_10_ reduction for mHP or “mHP vHP” treatments in presence of nisin (Fig. 3 and 7). This hypothesis is based on studies (38, 39) suggesting that HP can modify spore proteins, which facilitates nisin binding and, therefore, inactivation (HP: 500 MPa, 10 min, 50°C or 20°C, buffer or skim milk, 20 or 50 IU/mL nisin, *Bacillus* sp.) (38). The HP-induced sensitization to nisin seemed reversible or irreversible depending on the HP treatment temperature (39). As the heat treatment reduced the growth-inhibiting effect (Fig. 5 and 7), the mHP sensitization to nisin might be reversible by temperature. If and how proteins change their structure depends on the HP parameters (pressure, temperature, time, and sample matrix) (27, 57). Therefore, it is possible that vHP (550 MPa, 60°C, 2.5 min) might not have induced a spore modification, which facilitated nisin binding, at least not in the remaining ungerminated vHP superdormant spores. This is suggested by similar plate counts of vHP samples with or without nisin independent of the heat treatment (Fig. 3 and 5). Future studies are necessary to validate and further elucidate the proposed binding mechanisms of nisin to dormant or HP-treated spores. For future experiments, it should be kept in mind that the traditional plate count method with heat treatment might not be sufficient for the evaluation of the germination efficacy when nisin is present.

### Efficacy of combining different germination treatments

None of the tested HP treatment combinations could trigger the germination of all spores. The lowest germination efficacy was found for *B. subtilis* for mHP treatments (150 MPa, 37°C) without nisin and was comparable to previous literature for 150 MPa and 37°C (15, 58, 59). Treatments at a vHP of 550 MPa and 60°C were more effective. The measured reductions of 5–6 log_10_ units for vHP alone treatments of *B. subtilis* were also in the expected range for 550 MPa and 60°C (27, 59).

For *B. amyloliquefaciens*, germination induction at vHP was expected from a study at 550 MPa and 50°C and 0–30 min (60). The relatively low log_10_ reduction in our study (Fig. 3) confirms a high HP resistance of *B. amyloliquefaciens* spores compared to other spore-forming species (19, 40, 50). The HP resistance of the chosen *B. amyloliquefaciens* strain in our study might be related to its increased number of the *spoVA^2mob^* operon, which might confer pressure resistance (41). The *spoVA^2mob^* operon has been recently found to encode a protein (2Duf) that might be the main cause of the extreme wet heat resistance of strains carrying the *spoVA^2mob^* operon (61). The authors assumed the 2Duf protein and homologs to increase the wet heat resistance by reducing inner membrane permeability, which might protect membrane proteins (61). A decreased membrane fluidity seems to increase the resistance to a vHP of 500 MPa and 50°C (44). Therefore, it would be interesting to investigate the role of 2Duf and its homologs in vHP resistance.

As seen for both *Bacillus* species, increasing the HP treatment time increased the germination efficacy (Fig. 3 and 7; File S1: Fig. S3). Nevertheless, solely increasing the HP treatment time at ≤600 MPa and ≤80°C will unlikely achieve complete germination initiation of all species within <1 h. This is indicated by a tailing effect in germination or inactivation curves, i.e., decreasing treatment efficacy with increasing time (29, 52, 60, 62, 63).

In our study, germination stimulation of *B. subtilis* by mHP (150 MPa, 37°C, 5 min) seemed not strongly promoted by L-valine or L-alanine (Fig. 3, Fig. 7). These amino acids were tested for a possible synergism with mHP, as they activate GerA receptors in the nutrient germination pathway and GerA can be activated by mHP too in *B. subtilis* (50, 64). A small increase in the reduction of <1 log_10_ unit would have been expected from experiments at 150 MPa and 37°C with *B. subtilis* or *Bacillus licheniformis* and nutrients like alanine, the AGFK mix, or nutrient broth (45, 46). Various others reported a positive effect of a nutrient-rich matrix on the mHP (≤300 MPa) germination efficacy (37, 65–67). However, the effect of L-alanine on the *Bacillus* germination efficacy has also been shown to become negligible between 100 and 200 MPa at an unknown temperature and time (17). Hence, the increase in germination efficacy in our study might have been too small for clear detection by plate count or FCM (Fig. 3 and 4). As vHP (400–600 MPa) triggers germination mainly independently of nutrient germinant receptors (8, 18), a strong effect of >1 log_10_ unit of nutrients like valine on vHP (550 MPa, 60°C) germination seemed unlikely and was accordingly not observed (Fig. 3 and 7; File S1: Tables S1 and S2). No benefit of nutrients including milk has been suggested for vHP germination at ≥500 MPa for various *Bacillus* species (37, 38, 47). However, a nutrient-rich matrix, such as milk or crab meat, seemed beneficial in other studies on HP germination at 300–600 MPa of *B. subtilis* or *Bacillus cereus* (65, 68, 69).

Increased germination was not found in our study for the “mHP vHP” treatment compared to vHP alone (Fig. 3 and 7) even though the optimal pressure–temperature combination for mHP or vHP, respectively, for *B. subtilis* germination was applied (26, 43, 44). It suggests that an mHP (150 MPa, 37°C, 5 min) pre-treatment does not render the fraction, which is superdormant to vHP (550 MPa, 60°C, 2.5 min), more susceptible to the vHP germination trigger. Hence, if spores have a deficiency in DPA release under vHP, this deficiency seems not to be mitigated by an mHP germination stimulus, which can activate germinant receptors and subsequent DPA release through spoVA protein channels in the inner membrane (26). Or vice versa, vHP superdormant spores might also be poorly susceptible to mHP because mHP germination requires DPA release too. What the deficiency in DPA release under vHP means in structural or molecular terms is unclear because the vHP mechanism of DPA release is not fully understood. The release of DPA in vHP-germinating spores might be an effect of vHP directly on spoVA channels or an effect of vHP on the inner membrane (28). A “mHP vHP” combination of 100 MPa, 40°C, 30 min with 600 MPa, 40°C, 10 min has been previously tested for *B. subtilis* in phosphate buffer pH 7. Similarly to our study, complete germination could not be achieved by this combination, and the benefit of “mHP vHP” compared to vHP alone was not stated (70). In conclusion, two consecutive HP treatments at different HP germination conditions might unlikely prevent the occurrence of superdormant spores. The inability to germinate with nutrients or under HP might be partially explained by transient or reversible factors (15, 31, 32, 71). This was concluded from similar HP germination capacities of isolated nutrient superdormant spores and the corresponding dormant population used for superdormant spore isolation (71). Furthermore, ~60% of mHP superdormant spores, isolated after 20 min at 150 MPa and 37°C, initiated germination during a similar second mHP treatment (15). Little is known about how fast the superdormant state decays between two HP treatments. Short waiting times of 5–15 min at atmospheric pressure between two HP treatments did not clearly promote HP germination in previous studies (21, 45), but longer incubation times of ≥30 min at atmospheric pressure followed by a second HP treatment were assumed to increase the germination efficacy (72, 73). Considering that incubation at 37°C promotes nutrient germination, the increased log_10_ reductions for the treatments with incubation at 37°C for 6 h between two vHP treatments (Fig. 3 and 7) might underlie an increased nutrient germination of *B. subtilis*. However, an influence of the second vHP treatment on germination or inactivation of dormant spores by nisin cannot be excluded as the underlying cause for the increased log_10_ reduction.

The poor germination efficacy of *B. amyloliquefaciens*, compared to *B. subtilis*, for the ”Val Nis vHP 37°C vHP” treatment might be explained by poor HP germination and poor nutrient germination at 37°C. Previously, *B. amyloliquefaciens* germinated not with 10 mM L-valine at 37°C within 120 min while 10 mM L-alanine, for example, was more effective as seen by 20% germinated spores (74). Other free amino acids were present in the HP samples originating from the denatured milk solids (0.6 mg/mL in HP sample) in the commercial nisin product. However, the molar amino acid concentration seemed low (clearly <8 mM; 8 mM would assume all milk solid is free glycine). Therefore, germination with other amino acids in our samples at 37°C was likely ineffective for *B. amyloliquefaciens* too.

Repeated cycles of a short mild inactivation treatment (traditionally heat, could be HP) and a long incubation at atmospheric pressure have been evaluated for mild spore inactivation. The incubation usually lasts several hours and promotes nutrient germination of remaining dormant spores to inactivate them as germinated spores in the next inactivation treatment. This process is called “Tyndallization” (72, 75) and originates from the 19th century (75). The classic Tyndallization process has its limitations as (i) some spores could still resist the nutrient germination and inactivation treatments and (ii) some spore species could develop from germinated spores to growing, toxin-producing vegetative cells during the long incubation step (76–78). Hence, increasing the total treatment time by increasing the cycle number of vHP and incubation at atmospheric pressure without any growth-inhibiting factors seems neither a safe nor a time-efficient strategy for mild sterilization.

### Conclusion

The aim was to improve the spore germination–inactivation efficacy for the development of a mild sterilization process. Spore germination or inactivation treatments involving mHP (150 MPa, 37°C, 5 min), vHP (550 MPa, 60°C, 2.5 or 9 min), simple and complex nutrient germinants (L-valine, L-alanine, AGFK, and TSB medium), nisin, and incubation at atmospheric pressure were evaluated. The effect of these investigated parameters is shown in Fig. 7. The most effective treatment combination for *B. subtilis* spores involved nisin, a nutrient germinant (L-valine or TSB), a first vHP treatment (550 MPa, 60°C, 2.5 min) followed by incubation (atmospheric pressure, 37°C, 6 h), and a second vHP treatment (550 MPa, 60°C, 2.5 min). Despite combining different germination mechanisms, complete germination could not be achieved. *B. amyloliquefaciens* Technische Mikrobiologie Weihenstephan (TMW) 2.479 spores proved to be substantially more HP-resistant compared to *B. subtilis,* validating previous studies. Species variability affected spore germination or inactivation treatments and experimental methods, such as the sporulation conditions and spore washing.

To solve the complex problem of superdormancy, i.e., the resistance to a germination stimulus, solely increasing the HP treatment time, combining HP with one selected nutrient germinant like L-valine, or solely combining different HP levels and temperatures seems to be little effective. Even though nisin did seemingly not promote HP germination initiation, it might be beneficial to inhibit the growth of culturable HP-germinated or remaining ungerminated spores (38, 73, 79). Future studies might consider that nisin seemed not completely removable from spores by the chosen washing methods thereby affecting plate count (Fig. 7). It should be additionally considered that heat treatment can attenuate the reduction of culturable ungerminated spores caused by the combination of mHP and nisin (Fig. 7). Utilizing the findings regarding nisin to gain insights and potentially screen for other potent natural antimicrobials (lantibiotics) appears to be a promising approach for future applications.

Understanding the causes of HP resistance and superdormancy is key to the efficient development of a mild HP sterilization process. Especially, the time dependence of superdormancy seems worth further investigation to reveal mechanisms to overcome superdormancy and achieve complete germination. The spore behavior under pressure should be preferably studied *in situ*, as such studies are scarce (80, 81). *In situ* analysis allows the exclusion of effects of the post-HP conditions on germination (27). *Bacillus* and *Clostridium* species variability and the complex interplay of influencing factors (e.g., pressure, temperature, time, pH, and medium composition) must be considered in the future. HP treatment at a moderate temperature ≤80°C seems to only ensure effective mild bacterial spore inactivation in combination with additional germination promotors or preservation strategies. Evaluation of the combination of HP with other preservation strategies might be beneficial to lower the total processing intensity to better preserve the food quality or heat-sensitive objects. Our study contributes to the evaluation and development of mild preservation strategies in order to fulfill the consumer’s demand for safe and minimally processed food.

## MATERIALS AND METHODS

### Bacterial strains and spore preparation

The wild-type-like *B. subtilis* stain PS533 is a derivative of strain 168 and carries a plasmid with kanamycin resistance (82, 83). The *B. amyloliquefaciens* strain TMW 2.479 (Fad 82) was isolated from ropy bread (19).

PS533 spores were prepared as described by Zhang et al. (49) on Difco sporulation medium agar (pH 7.6) incubated for 4 days at 37°C. PS533 spores were >97% dormant spores as measured by FCM (method, see below) and confirmed by phase contrast microscopy (84). For sporulation of *B. amyloliquefaciens*, an overnight culture in TSB was streaked onto 2× SG agar [pH 7.0 (85)]. After 6 days at 30°C, agar plates were left at room temperature (24°C) for 4 days for lysis of vegetative cells. Spores were harvested by suspension in MilliQ water, and washed 5× on the day of harvest by centrifugation (6,000 × *g*, 10 min, 4°C) and twice per day in the first 7 days after harvest. The dormant spore purity was 89% as measured by FCM or 80%–90% under the phase contrast microscope. Impurities were mainly vegetative cells and few germinated spores. File S2 contains the detailed sporulation protocols.

PS533 and TMW 2.479 spores were stored protected from light at 4°C in MilliQ water and washed every 2 weeks. Two PS533 spore batches and one spore batch of TMW 2.479 were investigated. Vegetative *B. amyloliquefaciens* cells were removed prior to HP treatment by sonication (UP200H, Hielscher, sonotrode with 11 mm length and 7 mm diameter, estimated 100 W energy input in a 12.5 mL sample, 2 × 1 min treatment at 50% of maximal amplitude in ice bath, and 1-min waiting time on ice in-between) and 3× washing by centrifugation (6,000 × *g*, 10 min, 4°C).

### Choice of nisin concentration

To choose the nisin concentration for HP treatment, PS533 and TMW 2.479 spores at a concentration of 10^7^ CFU/mL were incubated in TSB with different final nisin concentrations (0.0025–2,500 IU/mL), the latter prepared by 1:10 serial dilution in a 96-well plate. The optical density was measured at 600 nm for 18 h at 37°C with shaking (plate reader Spark, Tecan, Switzerland) to measure the minimal inhibitory concentration (MIC) of nisin. Plates were further incubated at 37°C non-shaking for 2 days. Two technical replicates (wells) were measured on 3 days (*n* = 2 × 3) for each strain and each nisin concentration. The nisin stock contained 25,000 IU/mL commercial nisin [2.5% nisin A, 75% sodium chloride, and 22.5% denatured milk solids (% in wt/wt), nisin activity 1,052 IU/mg of the commercial product, REF 155839, MP Biomedicals, USA] dissolved in sterile-filtered 0.02 M HCl (0.2 µm, glass fiber cellulose acetate filter, Minisart, Sartorius). The nisin stock was stored at −20°C.

### High pressure treatment

Spores were treated at 150 MPa and 37°C (mHP) or 550 MPa and 60°C (vHP) to trigger germination or inactivation. An mHP treatment time of 5 min was chosen, as longer treatment times resulted in a little increase in the germination of *B. subtilis* relative to the increase in the total treatment time (15, 52). A vHP treatment time of 2.5 min was chosen as it resulted in 2 log_10_ units above the plate count detection limit for *B. subtilis* allowing a comparison to possibly more effective germination treatments. Spores were treated at concentrations of 10^8^–10^10^ CFU/mL in 50 mM N-(2-acetamido)-2-aminoethanesulfonic acid (ACES) buffer [pH 7.0, ThermoFisher, Germany, sterile-filtered using 0.2 µm polyethersulfone (PES) filter (Minisart, Sartorius)]. The final sample volume was 1.8 mL in polypropylene vials (vial N9 REF 702500, Macherey-Nagel, Germany) sealed using Nunc Cryoflex tubing (ThermoFisher, Germany). Nisin was added or replaced by sterile MilliQ water. For HP treatment with nutrients, the final L-valine (CAS 72-18-4) or L-alanine (CAS 56-41-7) concentration was 100 mM. TSB (File S2) was diluted by a factor of 2 due to other sample constituents. The HP machine (modified Model U111, Unipress, Poland) setup is described by Heydenreich et al. (27). A spore sample tube and a dummy tube without spores but 50 mM ACES buffer for temperature measurement during the HP treatment were cooled after sample preparation on ice (≥10 min) and for vHP treatments additionally at −20°C for 3 min. Both tubes were inserted into the HP machine at the exact same time. For the treatment “mHP vHP,” HP vessels were pressurized to 150 MPa and 37°C for 5 min, switched from one water bath (38.5°C) to another bath (61.4°C) within 25–37 s, and further pressurized to 550 MPa and 60°C. Exemplary pressure or temperature profiles of the HP treatments are shown in File S1: Fig. S5. Untreated dormant spores (NoHP controls) were kept on ice during the HP treatments of the other samples. Samples were kept on ice after HP for ≤15 min until further processing. For incubation between two vHP treatments, spores and a dummy tube were incubated immediately either at 37°C for 6 h or at 80°C for 10 min and then at 37°C for 6 h. These tubes were vortexed and pre-cooled for 15 min on ice and 3 min at −20°C before their second vHP treatment.

### Determination of spore germination or inactivation by spore washing, heat treatment, and plate count

The culturable spore fractions were determined by plate count. For nisin removal after HP treatment, 0.85–1 mL of the HP-treated or NoHP samples was washed by centrifugation (17,000 × *g*, 2 min, 4°C), supernatant removal with 200 µL pipet tips, and resuspension of the spore pellet in the initial volume (0.85–1 mL) of cold 50 mM ACES. Spores were kept on ice during waiting times. Centrifugation, supernatant removal, and resuspension were repeated three times. Spore samples were heat-treated at 80°C for 20 min in a water bath for the selection of culturable ungerminated spores by heat inactivation of germinated spores. An aliquot of a sample was diluted and plated before the heat treatment for comparison of plate count before and after heat treatment. Two dilution rows (in MilliQ, 1:10 dilution steps) were prepared for each sample, plated on tryptic soy agar, and incubated at 37°C. Colonies were counted in the next morning (after ~16 h) and recounted after 1 additional day. The spore concentration *N* (CFU/mL) or the reduction log_10_(*N*/*N*_0_) were calculated based on agar plates with 10–260 CFU as described in File S1: equations (1) and (2). The reference samples for the log_10_ reduction were NoHP controls of each day, reflecting the initial dormant spore concentration (*N*_0_) after washing and heat treatment (80°C, 20 min). Plates of undiluted samples with 1–10 CFU led to estimated log_10_ reductions due to a limited accuracy at low CFUs (86). Results are the means of experimental replicates (from HP treatment to plate count) performed on at least 3 different days (*n* ≥ 3). Statistical significance tests were not performed due to the small sample sizes (mostly *n* = 3).

### Analysis of germination and inactivation by FCM

Germination or inactivation was determined using FCM as described by Zhang et al. (49). Spores in sterile-filtered MilliQ water (10^7^ CFU/mL) were stained with final concentrations of 1.5 µM PI (CAS 25535-16-4, MP Biomedicals, USA) and 0.1 µM SYTO16 (Molecular Probes, Netherlands). PI indicates loss of membrane integrity and thus presumably inactivation (49, 87). SYTO16 stains germinated spores with an intact inner membrane and a (partially) degraded cortex (27). A number of 15,000 events at a rate of 6,000 ± 4,500 events/s were recorded in an LSR Fortessa cytometer [BD Biosciences, USA; excitation wavelength: 488 nm (SYTO16) or 561 nm (PI); emission collected through 530/30 band-pass filter (SYTO16, green fluorescence) or 610/20 band-pass filter (PI, red fluorescence)]. Measured controls and the gating scheme are depicted in File S1: Fig. S6 and S7. Spores were analyzed on the day of sample treatment or after freezing at −20°C for ≤2 weeks and thawing on ice.

## Data Availability

Raw data underlying the figures and table in this study are available via the ETH Zurich Research Collection (https://doi.org/10.3929/ethz-b-000645996).

## SUPPLEMENTAL MATERIAL

File S1 aem.02299-23-s0001.pdf Additional experimental data.

File S2 aem.02299-23-s0002.pdf Detailed sporulation protocols.

ASM does not own the copyrights to Supplemental Material that may be linked to, or accessed through, an article. The authors have granted ASM a non-exclusive, world-wide license to publish the Supplemental Material files. Please contact the corresponding author directly for reuse.
