# Supplementary material for: Strategies for effective high pressure germination or inactivation of Bacillus spores involving nisin
Source: Appl Environ Microbiol. 2024 Sep 23;90(10):e02299-23. doi: 10.1128/aem.02299-23 (PMC11505639; doi:10.1128/aem.02299-23)
Supplement: File S1 — Additional experimental data. [file aem.02299-23-s0001.pdf]

Content

1. Growth inhibition by different nisin concentrations 2
2. Effect of L-alanine and nisin on high pressure treatment of *B. subtilis* 3
3. Effect of L-valine or AGFK on high pressure treatment of *B. subtilis* 3
4. Effect of acidic washing on nisin removal 4
5. Effect of high pressure treatment time on *B. amyloliquefaciens* 5
6. High pressure treatment details 7
7. Flow cytometry gating strategy and controls 9
8. Calculation of spore concentration and log_{10} reduction 10

1. Growth inhibition by different nisin concentrations

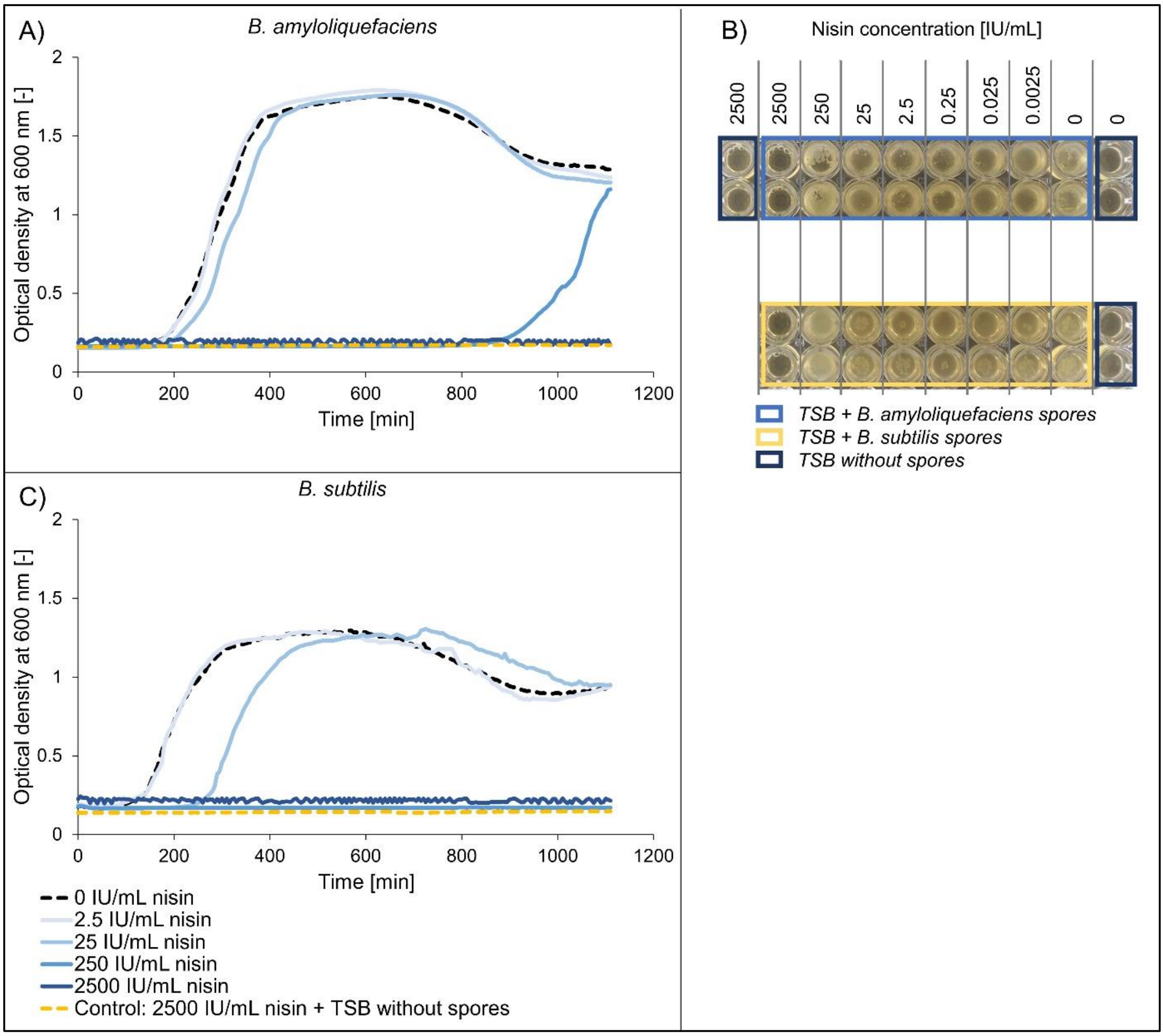

***Fig. S1**. Growth inhibition of Bacillus subtilis and Bacillus amyloliquefaciens by different nisin concentrations at atmospheric pressure. Spores at a concentration of 10^7 CFU/mL were incubated in TSB with different nisin concentrations (0 -2500 IU/mL) in a 96-well plate. The optical density was measured at 600 nm for 18 h at 37°C with shaking. Growth is indicated by a rise in the optical density in plots A) and C) where the optical density of a selected representative well is shown. Growth inhibition is indicated by a delay or lack of the signal rise. B): After additional 2 days at 37°C (non-shaking), growth was visible by the eye in terms of turbid medium in wells with spores and 0 - 250 IU/mL nisin. No turbidity (absence of growth) was found in wells with spores and 2500 IU/mL nisin or in control wells without spores but TSB and 0 or 2500 IU/mL nisin. A representative photo of the wells is depicted with 2 technical replicates (wells) of each sample type. The experiment was repeated on three different days.*

2. Effect of L-alanine and nisin on high pressure treatment of *B. subtilis*

Table S1. *Comparison of the effect of 100 mM L-alanine (Ala) or 100 mM L-valine (Val) and 2500 IU/mL nisin (Nis) on moderate-high-pressure-(mHP)-treated, very-high-pressure-(vHP)-treated, or dormant (NoHP) B. subtilis spores after washing, as analyzed by plate count.*

**Treatment**	**$\log_{10}$ (N/N$_0$)[c] reduction of cultivable dormant spores - after heat treatment[d]** (Mean ± SD, n≥3)
Ala Nis NoHP	-1.1 ± 0.1
Val Nis NoHP	-1.0 ± 0.1
Ala Nis mHP[a]	-2.5 ± 0.1
Val Nis mHP[a]	-2.4 ± 0.2
Ala Nis vHP[b]	-5.8 ± 0.2
Val Nis vHP[b]	-5.9 ± 0.2
vHP[b]	-5.7 ± 0.2

[a] 150 MPa, 37°C, 5 min.
[b] 550 MPa, 60°C, 2.5 min.
[c] Initial dormant spore concentration N_0 after heat treatment: 10^9 CFU/mL.
[d] Heat treatment at 80°C for 20 min.

3. Effect of L-valine or AGFK on high pressure treatment of *B. subtilis*

Table S2. *Effect of 100 mM L-valine (Val) or L-asparagine, D-glucose, D-fructose, and potassium chloride (AGFK; each 100 mM) on very-high-pressure-(vHP)-treated or dormant (NoHP) B. subtilis spores as analyzed by plate count without washing.*

**Treatment**	**$\log_{10}$ (N/N$_0$)[c] reduction of cultivable dormant spores - after heat treatment[d]** (Mean ± SD, n>3)
Val NoHP[a]	-0.1 ± 0.3
AGFK NoHP[a]	0.0 ± 0.1
vHP[b]	-5.1 ± 0.4
AGFK vHP[b]	-5.4 ± 0.3
Val vHP[b]	-5.6 ± 0.3

[a] Incubated at 60°C for 2.5 min as control treatment to vHP treatments.
[b] 550 MPa, 60°C, 2.5 min in ACES buffer. “vHP” samples were HP-treated as controls and analyzed on the same days as the “Val vHP” or “AGFK vHP” samples.
[c] Initial dormant spore concentration N_0 after heat treatment: 10^9 CFU/mL. Spore batch 2 was used for these experiments.
[d] Heat treatment at 80°C for 20 min. Washing before heat treatment was not necessary to remove the germinants L-valine or AGFK, as no further germination was induced between the HP treatment and the heat treatment, indicated by $\log_{10}$ reductions close to zero of the NoHP control.

4. Effect of acidic washing on nisin removal

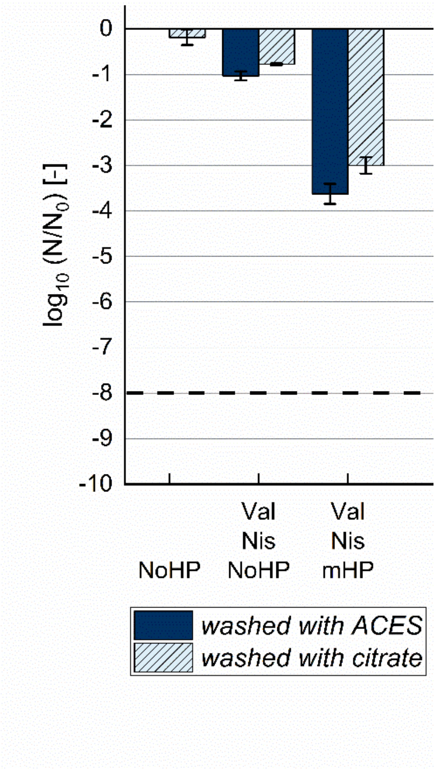

Fig. S2. *Effect of washing conditions on nisin removal and log_{10} reductions of cultivable dormant B. subtilis spores before heat treatment (N). Samples with or without 100 mM L-valine (Val) and 2500 IU/mL nisin (Nis) were moderate-high-pressure (mHP)-treated at 150 MPa and 37°C for 5 min or not mHP-treated but kept on ice (NoHP). Samples were washed by 3 cycles of centrifugation and supernatant exchange with 50mM ACES buffer pH 7.0 (standard procedure, "washed with ACES"). Removal of nisin was tested under acidic conditions by 2x resuspending the spore pellet in 100 mM citrate buffer (pH 2.5, 0.97 g citric acid CAS 77-92-9, 0.1 g sodium citrate dihydrate CAS 6132-04-3 in 50 mL MilliQ, sterilized with PES syringe filter) and 1x in 50 mM ACES buffer pH 7.0 after centrifugation (6000g, 10min, 4°C). To evaluate the effect of acidic washing on dormant spores alone, a NoHP sample without nisin and valine ("NoHP") is shown as control. Reductions relate to the initial dormant spore concentration (N_0) of 10^9 CFU/mL in the NoHP reference sample which was washed 3x with ACES and heat-treated for 20 min at 80°C. Error bars present standard deviations of independent experiments (n ≥ 3). - - - : Detection Limit.*

5. Effect of high pressure treatment time on *B. amyloliquefaciens*

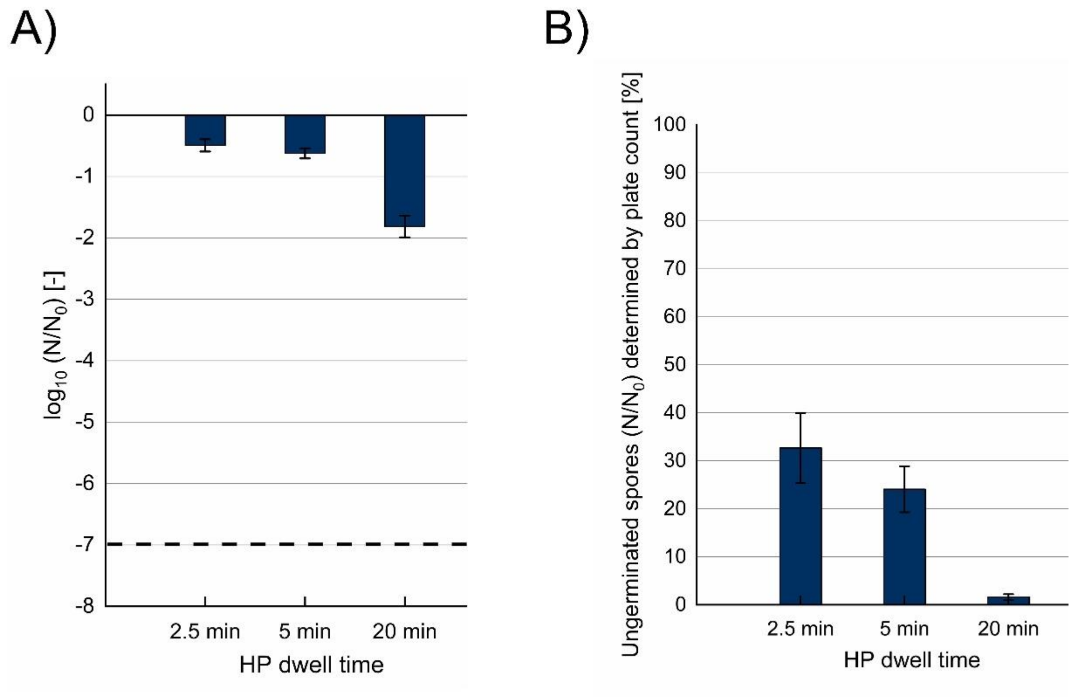

Fig. S3. *Reduction of cultivable dormant B. amyloliquefaciens spores (N) depending on the high pressure (HP) treatment time, i.e, the HP dwell time at 550 MPa and 60°C as determined by plate count and expressed as $log_{10}(N/N_0)$ (A) or percentage (N/N_0 *100) (B). Spores were HP-treated at an initial dormant spore concentration (N_0) of 10^8 CFU/mL and heat-treated (80°C, 20 min) before plating. Error bars present standard deviations of independent experiments ($n \geq 3$). - - - : Detection Limit.*

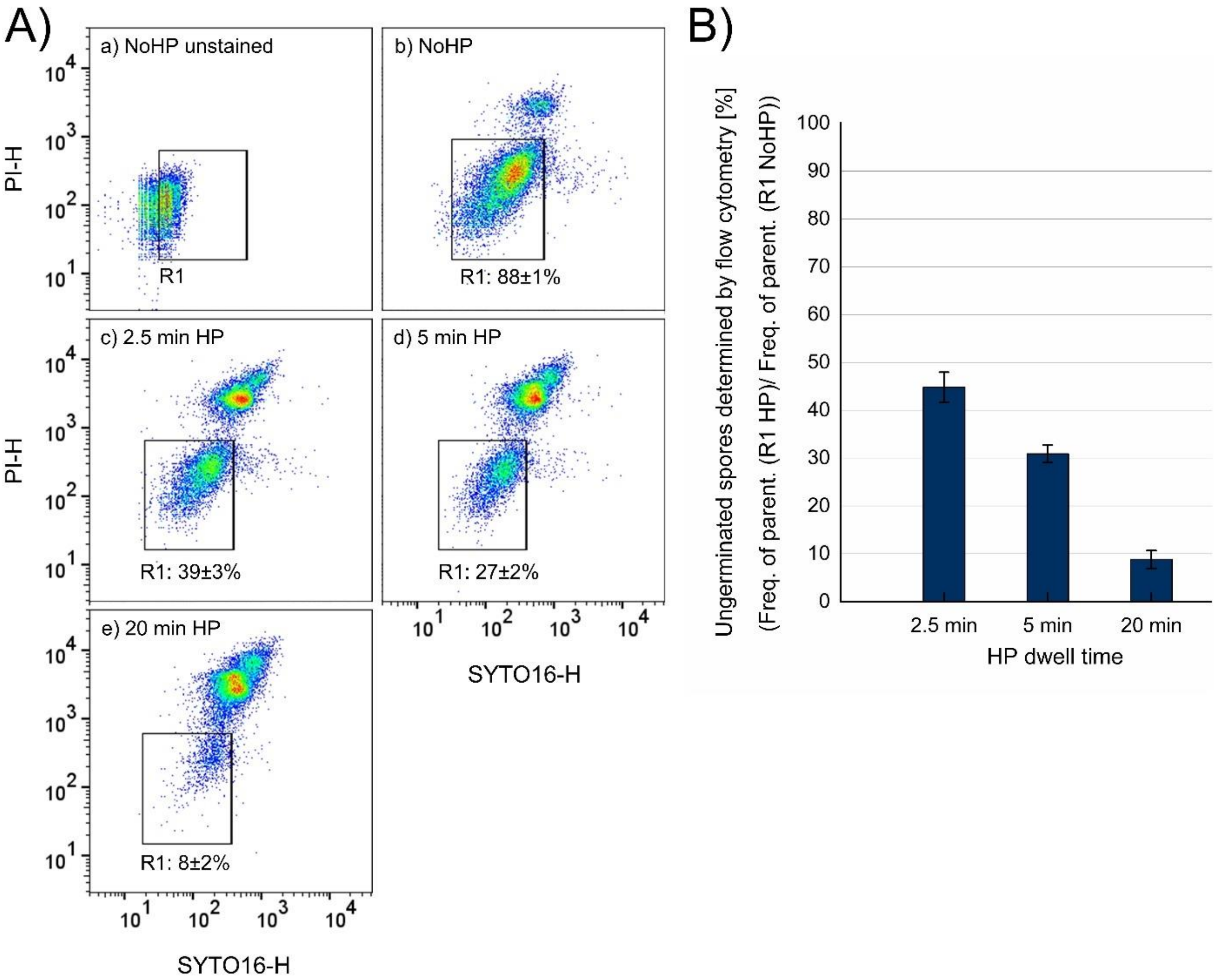

Fig. S4. *A): Flow cytometric quantification of ungerminated B. amyloliquefaciens spores depending on the on the high pressure (HP) treatment time, i.e., the HP dwell time at 550 MPa and 60°C. Samples were HP-treated and heat-treated (80°C, 20 min) before analysis. The samples were the same as those used for plate count analysis shown in Fig. S3. In the region R1 of certain fluorescence signal heights (-H) appears the spore subpopulations of propidium iodide (PI)- and SYTO16-negative, presumably ungerminated spores. The plot of the unstained untreated "NoHP" sample (a) is shown as control for autofluorescence of B. amyloliquefaciens spores. Percentages of events in R1 relative to all events in a plot are shown as mean ± standard deviation of experimental replicates from three different days. B): Normalized percentages of ungerminated spores corresponding to flow cytometry plots in A). Percentages of events in R1 ("Freq. of parent.") of HP- treated samples were normalized by the initial percentage of events in R1 of the untreated "NoHP" sample for each day (b) due to the poor dormant spore purity of the untreated samples (88±1%).* Values are expressed as *mean ± standard deviation of experimental replicates from three different days.*

6. High pressure treatment details

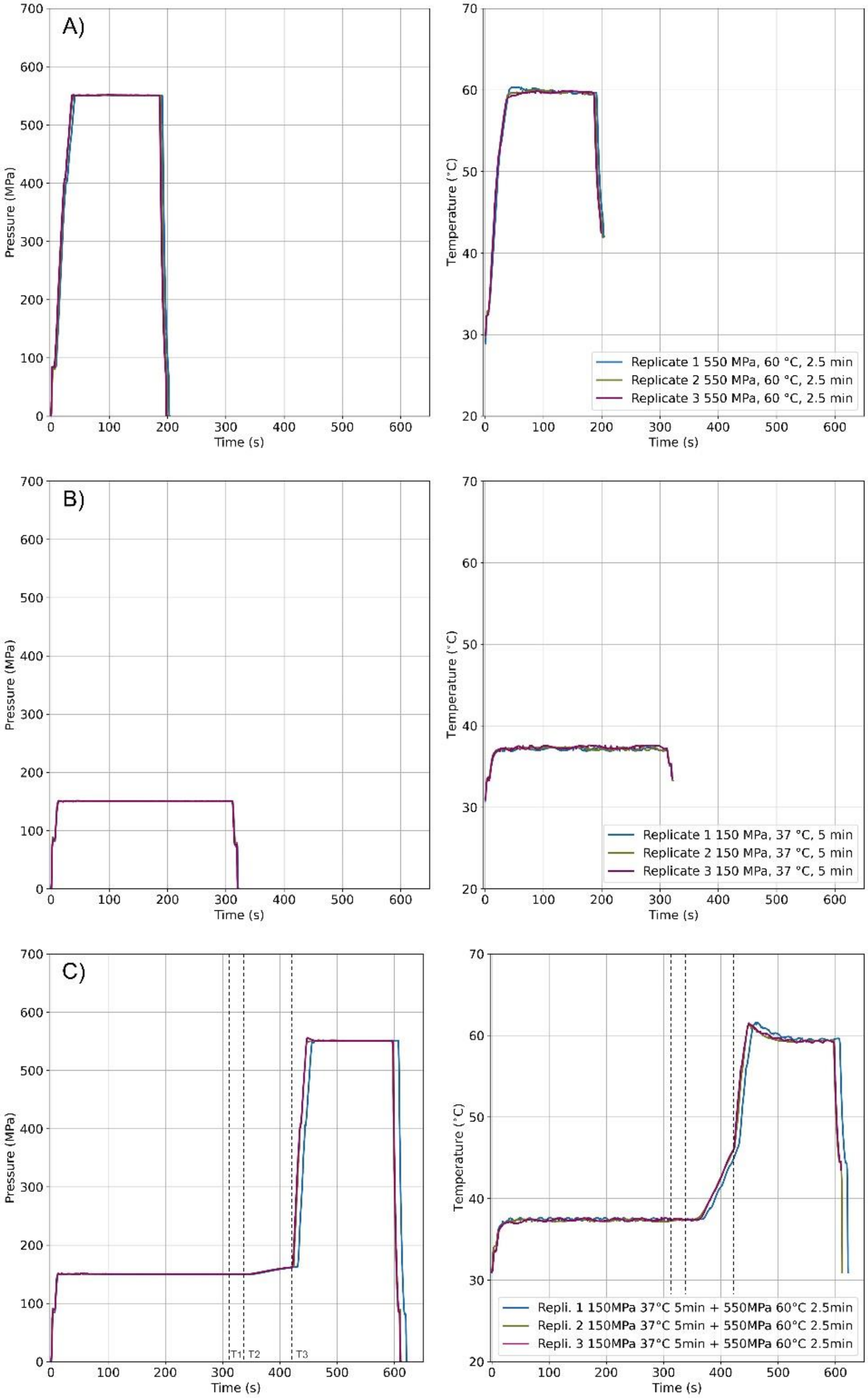

Fig. S5. *Representative pressure-time and temperature-time profiles for high pressure (HP) treatments in a modified Model U111 machine (Unipress, Warsaw, Poland) at A) 550 MPa and 60 °C (vHP) for 2.5 min, B) at 150 MPa and 37 °C (mHP) for 5 min or C) at "mHP+vHP" for 5 min and 2.5 min HP holding times, respectively. vHP treatments for 9 min showed similar profiles to vHP treatments of 2.5 min except 9 min pressure holding time and are therefore not depicted. Compression started when the dummy temperature reached 31.0°C and 29.5°C for mHP and vHP treatments, respectively, to reach the desired HP treatment temperatures without temperature overshoot due to adiabatic heating. For the "mHP+vHP" treatments, HP vessels were removed from the water bath 1 (38.5°C) at time T1, immersed in the water bath 2 (61.4°C) at time T2 and further compressed from 150 MPa to 550 MPa at time T3 at a dummy temperature of 46°C. Time points T1-T3 are shown as dashed lines in Fig. C) for replicate 3. For replicate 1, vessels were immersed in the water bath 2 (T2) 12 s later than for replicate 3 due to variations caused by manual shifting of the HP machine between water baths. The compression and decompression rates were approximately 15 MPa/s and 45 MPa/s, respectively.*

7. Flow cytometry gating strategy and controls

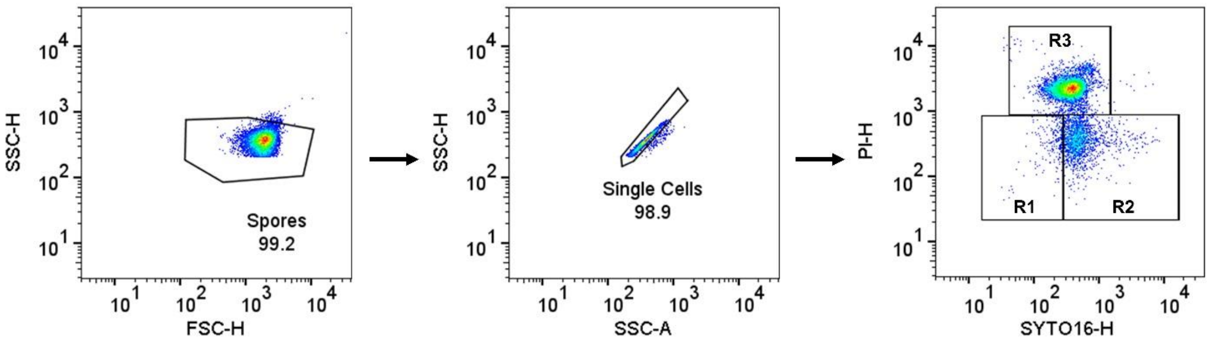

Fig. S6. *Gating strategy for flow cytometry measurements. Spore samples were stained with SYTO16 and PI. Events were gated for spores excluding background signals with high SSC-H values, then gated for single cells to exclude cell aggregates or multiple cells measured simultaneously, and then gated depending on the SYTO16 and PI fluorescence signals. The position of gates in SYTO16-H – PI-H plots was adapted to the absolute position of subpopulations due to variations in the PI- and SYTO16- signals between samples. However, the relative gate positions were the same for each sample. FSC-H: forward scatter signal height, SSC-H: side scatter signal height, SSC-A: side scatter signal area, PI-H/ SYTO16-H: propidium iodide (PI)/ SYTO16 fluorescence signal height.*

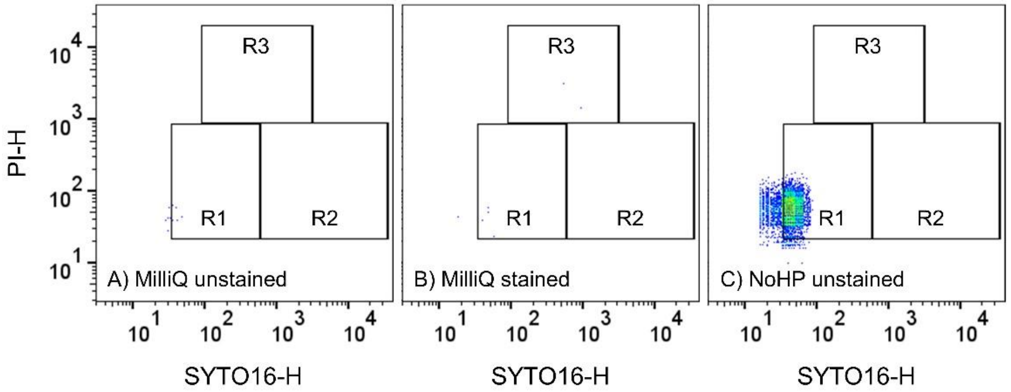

Fig. S7. *The following flow cytometry controls were measured every day: Filtered unstained MilliQ water (A), and MilliQ water stained with propidium iodide (PI) and SYTO16 (B) were recorded for the same time as the samples to monitor background signals; C): Untreated ("NoHP") and unstained B. subtilis spores were measured as control for autofluorescence. Representative plots are depicted.*

8. Calculation of spore concentration and log_{10} reduction

Equation 1. *Spore concentration based on plate counts.*

$$Spore\ concentration\ N\ \left[\frac{CFU}{mL}\right] = \frac{(CFU_1 + CFU_2) \div 2 \times dilution\ factor}{plated\ sample\ volume\ [mL]}$$

$CFU_{1,2}$:	colony forming units (counts) on agar plates of samples diluted by a certain factor; technical replicate 1 and 2 originated from two different dilution rows
Dilution factor:	factor equals 10^2 for a $1:10^2$ sample dilution, for example
Plated sample volume :	0.1 mL

Equation 2. *Logarithmic (log_{10}) reduction of cultivable spores based on spore concentration N (Eq.1).*

$$Log_{10}\ reduction\ [\,] = log_{10}\ \left[\frac{N}{N_0}\right]$$

N:	Culturable spore concentration in sample;<br>Culturable dormant spore concentration for heat-treated samples
N_0:	Initial culturable dormant spore concentration in reference sample;<br>Reference sample: non-HP-treated (NoHP) but kept on ice, washed 3x with ACES buffer, heat-treated at 80°C for 20 min
