# Supplementary material for: Strategies for effective high pressure germination or inactivation of Bacillus spores involving nisin
Source: Appl Environ Microbiol. 2024 Sep 23;90(10):e02299-23. doi: 10.1128/aem.02299-23 (PMC11505639; doi:10.1128/aem.02299-23)
Supplement: File S2 — Detailed sporulation protocols. [file aem.02299-23-s0002.pdf]

Sporulation Protocols for *B. subtilis* and *B. amyloliquefaciens*

Protocol A: Sporulation, harvest and washing of *B. subtilis*

Preparations

- Autoclaved, cold MilliQ water for spore washing (for 9 DSM agar plates at least 1 L)
- Liquid medium:

  Tryptic soy broth (TSB) + antibiotic (AB)
  Tryptic Soy Broth No. 2 (Sigma Aldrich) 30 g/ L
  Adjust volume to 1 liter with MilliQ and stir.
  Autoclave, cool down, and add the sterile-filtered (0.2 µm PES syringe filter, Sartorius) antibiotic kanamycin monosulfate (final conc. in TSB: 10 µg/mL, stored at -20°C) for strain PS533.

- Agar plates:

  Tryptic soy agar (TSA) + antibiotic (AB)
  Tryptic Soy Broth No. 2 (Sigma Aldrich) 30 g/L
  Agar 15 g/L
  Adjust volume to 1 liter with MilliQ and stir.
  Autoclave and add the sterile-filtered (0.2 µm PES syringe filter, Sartorius) antibiotic kanamycin monosulfate (final conc. in TSB: 10 µg/mL, stored at -20°C) for strain PS533 to hand-warm medium before plate pouring.

- **Sterile filtered mineral solutions** for DSM agar plates,
  sterile-filtered (0.1 µm or 0.2 µm PES syringe filter, Sartorius):
  1 M $Ca(NO_3)_2$ x 4 H_2O (e.g. Prepare: 2.36g + 10mL MilliQ)
  0.01 M $MnCl_2$ x 4 H_2O (e.g. Prepare: 0.198g + 100 mL MilliQ)
  1 mM $FeSO_4$ x 7 H_2O (e.g. Prepare: 0.0278g + 100 mL MilliQ)

  Note: these solutions cannot be stored for long time in the fridge, especially not the $FeSO_4$. Hence, solutions are prepared 1-2 days before DSM agar preparation.

Sporulation:

Tuesday, Day 0
Late afternoon, before leaving: Take a loop of frozen cryoculture and make a streak plate on a **TSA+AB plate**. Incubate at **37°C over night.** Growing *B. subtilis* on a TSA plate already on Tue allows to check growth behavior of mutant strains, sterility of medium and activity of antibiotics. These plates can serve as backup when stored in the fridge.

Control 1: Incubate an empty plate as sterility control.
Control 2: Streak out a *B. subtilis* strain without antibiotic resistance on a plate (e.g. PS832 = same strain as PS533 but without AB resistance) (no growth the next day if AB is active).

Wednesday, Day 1
Morning: Prepare fresh **DSM medium** and autoclave it. Don't forget to add the salt solutions after autoclaving. No antibiotics are used as sporulation is so energy consuming, that we don't want to give an additional hurdle. Produce DSM agar plates in square plates (125 x 125 x 20mm). Pour plates equally thick (1 cm). Dry agar plates without lid in a sterile environment until the condensed water has evaporated

from the lids. Leave the plates at room temperature in a loosely closed plastic bag.

Difco Sporulation Medium (DSM) (1L yields approx. 8-9 square plates)

Difco Nutrient Broth (BD 234000)	*8g/L*
KCl	*1g/L*
$MgSO_4 \cdot 7H_2O$	*0.12g/L*

Adjust volume to 1L with MilliQ water. Set pH to 7.60 with 1M NaOH. Fill in 1L bottles and add magnetic stirrer. Add:

Agar	*15g /1L bottle*

Autoclave and allow to cool to 50°C. Just prior to use, add the sterile-filtered solutions from above:

1 M $Ca(NO_3)_2$	*x 4 H_2O*	*1 mL/1L bottle*
0.01 M $MnCl_2$	*x 4 H_2O*	*1 mL/1L bottle*
1 mM $FeSO_4$	*x 7 H_2O*	*1 mL/1L bottle*

Late afternoon, before leaving: Take a loop of frozen cryoculture and make a streak plate on a **TSA+AB plate**. Incubate at **37°C over night.** Growing *B. subtilis* on a TSA plate prior to growth in TSB allows to control for contamination and improves bacterial fitness.

Controls as on Day 0. Control 2 can be omitted if the same batch of antibiotics is used.

Thursday, Day 2

Morning: Put TSA+AB plate at room temperature in the morning to avoid that single colonies grow together.

Around lunchtime: Start liquid culture. Make sure the media is not freezing cold to avoid cold shock. Pick one colony and add it to **10 ml TSB+AB** liquid media in a 50 mL centrifugation tube for good aeration. Don't close the lid entirely (aerobic growth). Incubate at **37°C, 250 rpm**.

Control 1: Tube with only media to control sterility of media (no turbidity the next day).
Control 2: Tube with a *B. subtilis* strain without antibiotic resistance (e.g. PS832 = same strain as PS533 but without AB resistance) (no turbidity the next day if AB is active).
Note: Control 2 can be omitted if the same batch of antibiotics as for the TSA plates is used.

Afternoon: Put empty DSM plates (without bag) at 37°C over-night before usage on the next day.

Friday, Day 3

Early morning: Refresh liquid culture: Take 1mL of the liquid overnight culture and inoculate 9 mL of fresh TSB+AB in a new 50 mL tube. Make sure to do 3-4 of these tubes depending on the amount you need for inoculation of DSM plates. Don't close the lid entirely (aerobic growth). Incubate at **37°C, 250 rpm**.

Controls: As Day 2.

Morning: Take DSM plates out of incubator and let them dry at room temperature with open lid under the sterile bench until condensed water has evaporated.

After ca. 1-4h: When OD_{600} of the culture reaches **1.8** - **2** (measure 1mL), spread 300µl of culture on DSM plates. Wrap stacks of 4 plates in a plastic bag to avoid that plates dry out (use clean ~20L biohazard bags and don't close them completely airtight, just wrap open end around). Incubate plates upside down at **37°C**.

Day 4-7

Check under the phase contrast microscope 2-3 days after inoculation of DSM plates, then daily, if sporulation is complete and spores are released from the sporangia. Complete release usually takes 2 – 7 days, depending on the strain. Take care when removing plates from 37°C, only take one plate at a time

and work fast, put plate immediately back so sporulation takes place at constant temperature for all plates. Dormant spores are phase bright and appear first inside the mother cell which then lyses and releases the spore. Unreleased forespores are impossible to clean up. Released spores may start to germinate. Germinated spores are round and phase dark. If spores start to germinate, harvest spores quickly! When the bright-phase spore percentage is higher than 90% and the surface of the agar plate looks shiny, spores can be harvested. This is typically on Tuesday (day 7) for strain PS533.

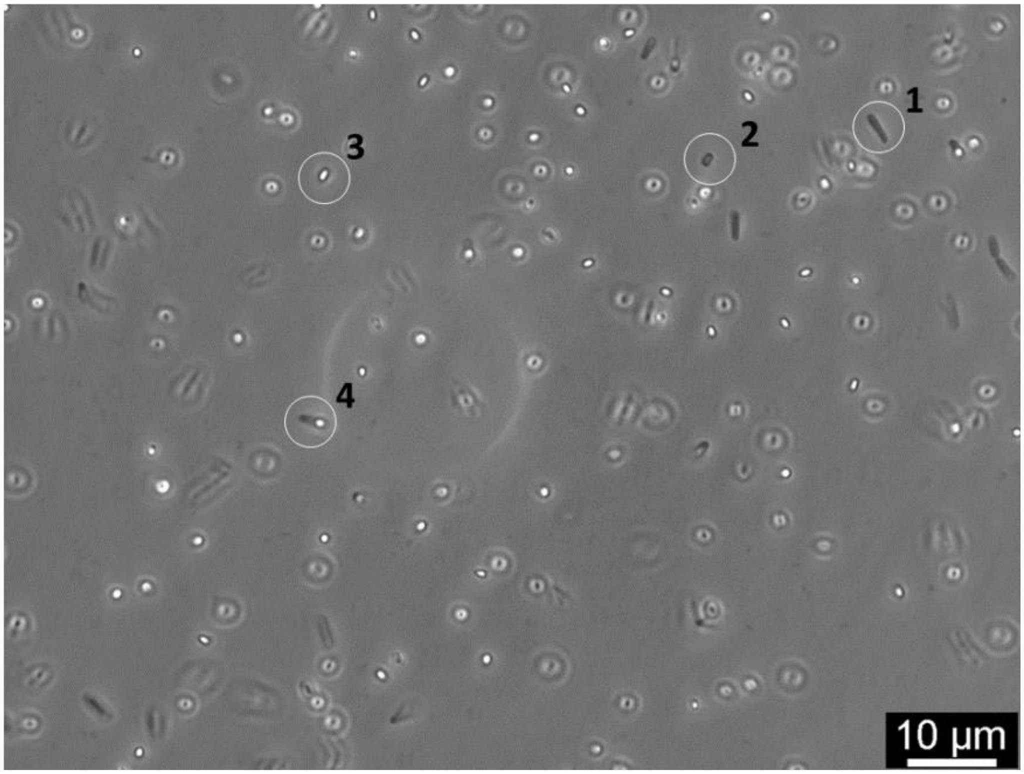

Fig. S1*. Phase contrast microscopy of B. subtilis cell types one day before harvesting. Vegetative cell (1), germinated spore (2), dormant spore (3), mother cell with a forespore (4).*

Spore harvesting:

Precool centrifuge to 4°C!

1. Add 10 mL of <u>cold</u> sterile MilliQ (**4°C, ensure it stays cold**) to a DSM plate (measure with 50mL falcon).
2. Re-suspend grown colonies containing spores with a sterile spreading stick. Avoid breaking the agar (we don't want to transfer any nutrients).
3. Transfer the suspension from the plates to a 50 mL centrifugation tube with a sterile 10 mL pipette. Keep the tube on ice.
4. Repeat steps 1-3 two more times until the agar of the plates becomes transparent again (collect as many spores as possible in the 50 mL centrifuge tube).
5. Vortex and store tube on ice until washing.

➔ Repeat for all DSM plates.

Spore washing:

1. Centrifuge spore suspension at **6’000 g, 10 min, 4°C** and discard the supernatant.
2. Re-suspend pellet in 25mL sterile MilliQ and vortex well or shake by hand. Hurry as the samples are not cooled during mixing.

On the day of harvest:

3. Repeat steps 1-2 at least four more times.
   This serves to remove cell debris from lysed cells who had released spores and to remove molecules that may potentiate germination- Check under the microscope that harvested samples are >90% pure dormant spores. Otherwise keep washing.
4. Label falcon tubes properly with *strain name*, *date* (of harvesting) and *Nr. for tubes* 1,2,3...Also make a clear line for the upper level of the liquid in the falcon (this is important to maintain the same concentration of spores when washing). Put transparent adhesive tape over the labeling as excessive washing can remove labeling.
5. Store the resuspended spores, protected from light, at 4°C.

For 1 week after harvesting:

Repeat steps 1-2 to wash daily once in the morning and afternoon for the following 7d. If spores have been washed already 10 times in total, it is ok to not wash them during the weekend.

Spore batch pooling:

1. Check purity of each tube under the microscope (min. 95% phase-bright spores).
2. Pool all the spores by pouring the spore suspensions in a sterile 1L flask with magnetic stirrer. Mix well and redistribute suspensions à 25mL to new 50mL tubes using as sterile pipet. Pooling reduces heterogeneity in spore properties between tubes of the same spore batch. Label each tube properly.

Spore batch storage:

- Store spores protected from light at 4°C.
- After the first week of intensive washing and batch pooling, wash spores every 2-3 weeks once. When washing, always fill up to the marked line with MilliQ to maintain approx. concentration!
- The spore concentration depends on the B. subtilis strain, for PS533 it is usually 10^9 CFU/mL.
- Check dormant spore purity over time, e.g. by phase-contrast microscopy.
- When working with spores, keep them on ice to minimize spontaneous germination.

Protocol B: Sporulation, harvest and washing of *B. amyloliquefaciens*

Using the same sporulation protocol for *B. subtilis* for sporulation of B. amyloliquefaciens TMW 2.479, the desired spore purity could not be achieved due to remaining vegetative cells. Vegetative cells of *B. subtilis* can be removed by extensive washing with MilliQ water, as described above. The sporulation protocol for *B. amyloliquefaciens* was adapted using 2xSG sporulation medium and incubation of 2xSG agar plates at room temperature for lysis of vegetative cells. *B. amyloliquefaciens* spores produced with this adapted protocol did not reach the usually desired purity of > 95% and formed agglomerates of >100 spores upon storage in MilliQ water.

Preparations

- Prepare **MilliQ** water, **TSB** and **TSA** agar plates similar to as described for *B. subtilis*. However, prepare TSB and TSA without antibiotics for the strain TMW 2.479.
- **Sterile filtered mineral solutions** for **2x SG agar** plates,
  sterile-filtered (0.1 µm or 0.2 µm PES syringe filter, Sartorius):

1 M $Ca(NO_3)_2$	*x 4 H_2O*	*(e.g. Prepare: 2.36g + 10mL MilliQ)*
***0.1 M** $MnCl_2$*	*x 4 H_2O*	*(e.g. Prepare: 0.198g + 10 mL MilliQ)*
1 mM $FeSO_4$	*x 7 H_2O*	*(e.g. Prepare: 0.0278g + 100 mL MilliQ)*
25% (250g/L) Glucose		*(Prepare: 2.5g + 10 ml MilliQ). Warm glucose at 50°C to accelerate dissolution.*

Note: these solutions cannot be stored for long time in the fridge, especially not the $FeSO_4$. Hence, solutions are prepared 1-2 days before usage for agar preparation.

Sporulation:

Tuesday, Day 0

Inoculate and incubate TSA plates as described for *B. subtilis*. Control 2 can be omitted, as no antibiotics are used for *B. amyloliquefaciens*.

Wednesday, Day 1

Morning: Prepare fresh **2xSG medium** and autoclave it. Don't forget to add the salt solutions after autoclaving. No antibiotics are used as sporulation is so energy consuming, that we don't want to give an additional hurdle. Produce agar plates in square plates (125 x 125 x 20mm). Pour plates equally thick (1 cm). Dry agar plates without lid in a sterile environment until the condensed water has evaporated from the lids. Leave the plates at room temperature in a loosely closed plastic bag.

2xSG Sporulation Medium (1L yields approx. 8-9 square plates)

Adapted from (Nicholson and Setlow, 1990)

Difco Nutrient Broth (BD 234000)	***16** g/L*
KCl	***2** g/L*
$MgSO_4 \cdot 7H_2O$	***0.5** g/L*

*Adjust volume to 1L with MilliQ water. Set pH to **7.00** with 1M NaOH. Fill in 1L bottles and add magnetic stirrer. Add:*

Agar	***17g** /1L bottle*

Autoclave and allow to cool to 50°C. Just prior to use, add the sterile-filtered solutions from above:

1 M $Ca(NO_3)_2$	*x 4 H_2O*	*1 mL/1L bottle*
***0.1 M** $MnCl_2$*	*x 4 H_2O*	*1 mL/1L bottle*
1 mM $FeSO_4$	*x 7 H_2O*	*1 mL/1L bottle*
25% Glucose		***4 mL**/1L bottle*

Late afternoon, before leaving: Inoculate and incubate TSA plates as described for *B. subtilis*. Use same controls as on day 0.

Thursday, Day 2

Morning: Put TSA plates at room temperature to avoid that single colonies grow together.

Around lunchtime: Start liquid culture in TSB without antibiotic as described for *B. subtilis*. Control 2 can be omitted.

Afternoon: Put 2xSG plates (without bag) at 37°C over-night before usage on the next day.

Friday, Day 3

Early morning: Refresh liquid culture as described for *B. subtilis*: Use TSB without antibiotic.

Morning: Take 2xSG plates out of incubator and let them dry at room temperature with open lid under the sterile bench until condensed water has evaporated.

After ca. 1-4h: When OD_{600} of the culture reaches **1.8** - **2** (measure 1mL), spread 300µl of culture on 2xSG plates. Wrap stacks of 4 plates in a plastic bag to avoid that plates dry out (use clean ~20L biohazard bags and don't close them completely airtight, just wrap open end around). Incubate plates upside down at **30°C**.

Day 4-9

Check under the phase contrast microscope after inoculation of 2xSG plates daily when sporulation is complete and spores are released, as described for *B. subtilis*.

When bright-phase spore percentage is around 90% (typically Wednesday (day 8) or Thursday morning (day 9) for strain TMW 2.479), the agar plates are left without plastic bag at room temperature (24°C) on the bench.

Day 10-13

Check under the phase contrast microscope if mother cells have lysed but dormant spores have not germinated.

When bright-phase spore percentage is > 90% and the surface of the agar plate looks shiny, spores can be harvested.

Spore harvesting, Spore washing, Spore batch pooling, Spore batch storage

Follow procedure as described for *B. subtilis*.
